# Genome-wide dissection reveals diverse pathogenic roles of bacterial Tc toxins

**DOI:** 10.1371/journal.ppat.1009102

**Published:** 2021-02-04

**Authors:** Nan Song, Lihong Chen, Zhemin Zhou, Xingmei Ren, Bo Liu, Siyu Zhou, Caihong Wang, Yun Wu, Nicholas R. Waterfield, Jian Yang, Guowei Yang

**Affiliations:** 1 Beijing Institute of Tropical Medicine, Beijing, China; 2 Emergency and Critical Care Center, Beijing Friendship Hospital, Capital Medical University, Beijing, China; 3 NHC Key Laboratory of Systems Biology of Pathogens, Institute of Pathogen Biology, Chinese Academy of Medical Sciences and Peking Union Medical College, Beijing, China; 4 Warwick Medical School, Warwick University, Coventry, United Kingdom; University of Toronto, CANADA

## Abstract

Tc toxins were originally identified in entomopathogenic bacteria, which are important as biological pest control agents. Tc toxins are heteromeric exotoxins composed of three subunit types, TcA, TcB, and TcC. The C-terminal portion of the TcC protein encodes the actual toxic domain, which is translocated into host cells by an injectosome nanomachine comprising the other subunits. Currently the pathogenic roles and distribution of Tc toxins among different bacterial genera remain unclear. Here we have performed a comprehensive genome-wide analysis, and established a database that includes 1,608 identified Tc loci containing 2,528 TcC proteins in 1,421 Gram-negative and positive bacterial genomes. Our findings indicate that TcCs conform to the architecture of typical polymorphic toxins, with C-terminal hypervariable regions (HVR) encoding more than 100 different classes of putative toxic domains, most of which have not been previously recognized. Based on further analysis of Tc loci in the genomes of all *Salmonella* and *Yersinia* strains in EnteroBase, a “two-level” evolutionary dynamics scenario is proposed for TcC homologues. This scenario implies that the conserved TcC RHS core domain plays a critical role in the taxonomical specific distribution of TcC HVRs. This study provides an extensive resource for the future development of Tc toxins as valuable agrochemical tools. It furthermore implies that Tc proteins, which are encoded by a wide range of pathogens, represent an important versatile toxin superfamily with diverse pathogenic mechanisms.

## Author summary

Entomopathogenic bacteria deploy a range of toxins to combat their insect hosts. The Tc toxins were first identified in *Photorhabdus* as having potent oral toxicity to insects, with a mode of action distinct from the well-studied *Bacillus thuringiensis* Cry toxins. As such the Tc toxins have been considered as potential candidates for novel crop protection strategies. This could mitigate against the potential risks of pest insects developing resistance to the traditionally used Cry toxin-based systems. To date, the generality of diverse Tc toxins and their related pathogenic roles has remained mainly obscure. Our analysis has showed Tc toxins are widely distributed among Gram-negative and positive bacterial genomes. A database was constructed including thousands of Tc loci with hundreds of different putative TcC toxic domains, any one of which might represent candidates for the development of future pest control systems. Moreover, the findings of this study are of wider significance because Tc toxin homologues have been shown to be encoded by a range of human pathogens. These include *Salmonella* and *Yersinia*, suggesting their potential roles in human infectious diseases. Together, this study describes the characteristics and distribution of Tc toxins among diverse bacterial genera, and provides a new insight into their roles in different pathogenesis mechanisms. This study also describes findings of potential importance to their development as tools for biotechnological applications.

## Introduction

Selection pressures in complex environments have driven bacteria to evolve numerous strategies to transfer proteins into the cells of diverse eukaryotic and/or prokaryotic organisms [1]. Collectively known as “effectors”, these proteins are involved in mediating various interactions, including symbiosis, pathogenicity and competition with other microbes [2–4]. Several specialized nanomachines have been described which are used to deliver specific effectors into target cells, including the well-studied type III and VI secretion systems and extracellular contractile injection systems [5–8]. These effector proteins have been shown to play key roles in the lifecycles of prokaryotes in a diversity of environments.

Members of the polymorphic toxin systems (PTs) produced by bacteria are widespread tools used for interspecies bacterial competition [9,10]. The first described PTs, causing contact-dependent growth inhibition (CDI) was the CdiB/CdiA two-partner secretion (TPS) system. It requires a small immunity protein (CdiI) to prevent self-intoxication and the sequences of the C-termini of CdiA proteins exhibit high variability [11,12]. Another example of PTs is the Rearrangement hot spot (Rhs) repeat proteins, which are widespread in both Gram-negative and positive bacteria with the similar features as the CdiBAI system [13,14]. There are many more T6SS effectors which exhibit polymorphic toxin characteristics. The N-terminal regions of “evolved” VgrG proteins contain two conserved phage domains, which are fused to polymorphic C-terminal domains, facilitating various toxic activities against target cells [15,16]. In general, PTs include a toxic protein, which has bipartite architecture, with a conserved N-terminal region fused to variable C-terminal toxic domain and a specific immunity protein which is required to confer self-protection against the toxin.

First identified in *Photorhabdus luminescens*, the insecticidal Tc toxins, are multiprotein complexes composed of TcA, TcB and TcC subunits, used by these bacteria to kill their insect hosts, which are considered as potential alternative candidates of the *Bacillus thuringiensis* Crystal-toxin crop protection technology [17,18]. Homologues have since been discovered encoded in the genomes of many other species of entomopathogenic bacteria [19–23]. For example, *Yersinia entomophaga* secretes an ABC toxin (YenTc) which exhibits oral toxicity towards coleopteran species [24]. Fuchs *et al*. investigated the biological role of the *tc* genes in *Yersinia* spp., and suggested the presence of the Tc toxin correlates with a higher larvae toxicity of *yersiniae* towards *Manduca sexta* [25]. Moreover, though not toxic to fleas, *Y*. *pestis* Tc proteins can inhibit phagocytosis by mouse polymorphonuclear leukocyte to subvert the innate immune response [26].

In a typical Tc toxin complex, the TcA subunit forms a homo-pentamer, which is responsible for host cell receptor binding, membrane penetration and toxic polypeptide translocation [27,28]. The TcB and TcC subunits form a heterodimer, with a cocoon-like structure, which interacts with the TcA pentamer to facilitate translocation of a toxic polypeptide domain into the host cell [29,30]. The C-termini of several previously described TcC subunits represent hypervariable regions (HVRs) typically encoding known toxin domains, with examples including two ADP-ribosyltransferases, TccC3 and TccC5 [31]. Lang et al. reported that TccC3 can ADP-ribosylate actin at Thr148, leading to F-actin aggregation, which further inhibits cellular functions, such as phagocytosis. TccC5 can ADP-ribosylate Rho GTPases at Gln63 and Gln61, thus inhibiting the GTP hydrolysis activity [31]. Despite this knowledge, the distribution and versatility of Tc toxins across different bacterial genera remains unclear.

Here, we have combined both bioinformatic and experimental approaches to comprehensively analyze Tc toxins in order to provide a detailed understanding of this important toxin superfamily. We have used a protein profile and genomic context-based method to scan currently available bacterial genomes and generate a Tc database (dbTC; http://www.mgc.ac.cn/dbTC/), which includes 1,608 Tc loci and 2,528 TcC proteins. Our findings reveal that, as a new PTs, TcC HVRs encode hundreds of different toxic domains, including over 100 as yet uncharacterized domains, which could be used to mediate a bewildering array of potential interactions between bacteria and eukaryotic cells. We performed a higher resolution analysis of 7,024 and 1,940 TcC proteins identified in genomes of *Salmonella* and *Yersinia* strains from EnteroBase. A two-level evolutionary process was proposed to explain the taxonomical specific distribution pattern of TcC HVRs. Our findings imply that, as a widely distributed bacterial toxin system, Tc toxins are not only candidates for further pest control development but also likely to play fundamental roles in the lifecycles of many bacterial species, especially in their interactions with eukaryotic hosts.

## Results

### Identification and distribution of Tc toxins

**Fig 1:**
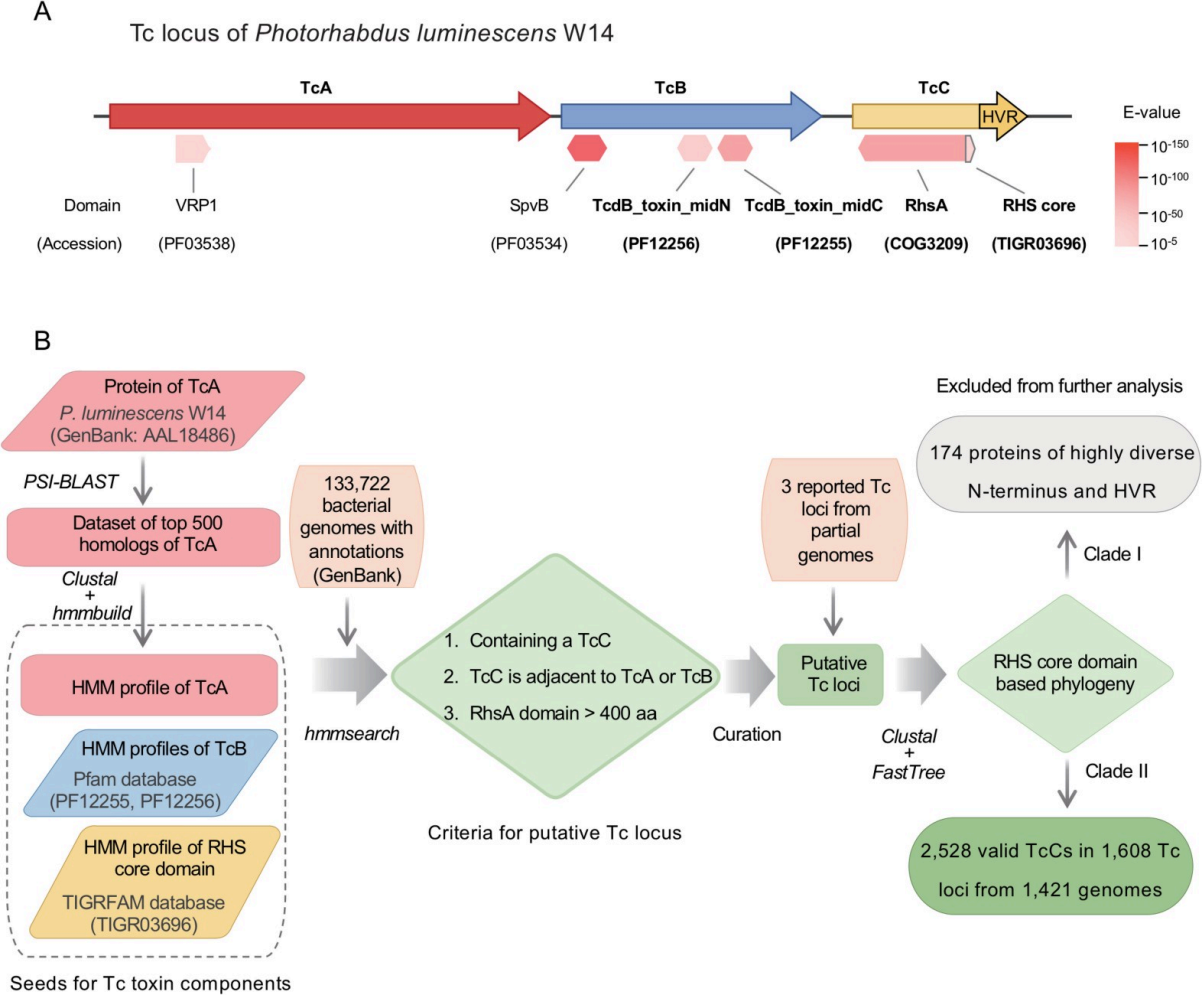
Identification of Tc toxins among bacterial genomes. (A) The genetic organization of a prototypical Tc locus from *Photorhabdus luminescens* W14. Arrows represent the three Tc subunits: TcA (red), TcB (blue) and TcC (yellow). The TcC HVR is colored in dark yellow. Conserved domains identified by CDD database (as of Jan, 2019) are shown below as hexagons (complete domain) or a pentagon (partial domain), with color coded to the RPS-BLAST E-values (to scale). The VRP1 and SpvB domains are specifically related to proteins encoded on a *Salmonella* virulence plasmid, thus were not used in this study. The domains used for screening are marked in bold. (B) The schematic workflow for the identification of Tc toxins from complete/draft bacterial genomes.

To characterize the diversity and distribution of Tc toxins, we attempted to identify all potential homologues from a total of 133,722 publicly available complete and draft bacterial genomes in the Reference Sequence (RefSeq) collection of GenBank. Our screening approach is based on information regarding common features of functionally well-studied Tc loci [28,32,33]. The three subunits of a prototypical Tc (TcA, TcB and TcC) were identified based on known conserved domains and/or hidden Markov model (HMM) profiles of protein alignments (Fig 1A).

N-termini of TcC proteins encode RhsA and RHS core domains, which are also highly conserved in Rhs proteins [13]. In order to exclude Rhs proteins, an empirical criterion was applied to our screening protocol which ignored any proteins containing an Rhs domain that is not encoded in the genomic neighborhood (<10 kb) to *tcA* or *tcB* gene homologues. This is because functional studies of characterized Tcs show that TcC subunit need to bind with TcB, which together then associate with a TcA pentamer to become toxic [28]. An additional criterion we applied was that putative TcC proteins should have a relatively complete RhsA domain (>400 aa), the region responsible for binding to TcB. This combined protein HMM profile and genomic context-based approach for the initial identification of *bona fide* TcC proteins is summarized in Fig 1B.

After a comprehensive screening process, supported with careful manual curation, a total of 2,702 TcC protein candidates were identified. To further characterize these TcC candidates, we produced a phylogenetic tree of these 2,702 proteins, based on the RHS core domain (S1A Fig). The tree forms two distinct clades with different characteristics. A minor clade (Clade I) consists of 174 highly diverse members in terms of protein size, at least 10 of which harbor a PAAR domain at the beginning of their N-termini (S1B Fig). This domain was also identified in T6SS related Rhs proteins [34]. In contrast, the major clade (Clade II) includes 2,528 proteins which generally exhibit similar sizes for both their N-terminal domains and C-terminal HVRs. Moreover, we observed a significant difference between the sequences of the RHS core domains in members of these two clades, despite both possessing the conserved PxxxxDxxG residues (S1C Fig). Therefore, to avoid potential contamination of the TcC dataset with Rhs-related proteins, the 174 proteins in Clade I were excluded from our subsequent analyses. Thus, the final curated collection contains 2,528 putative TcCs within 1,608 loci from 1,421 bacterial genomes.

**Fig 2:**
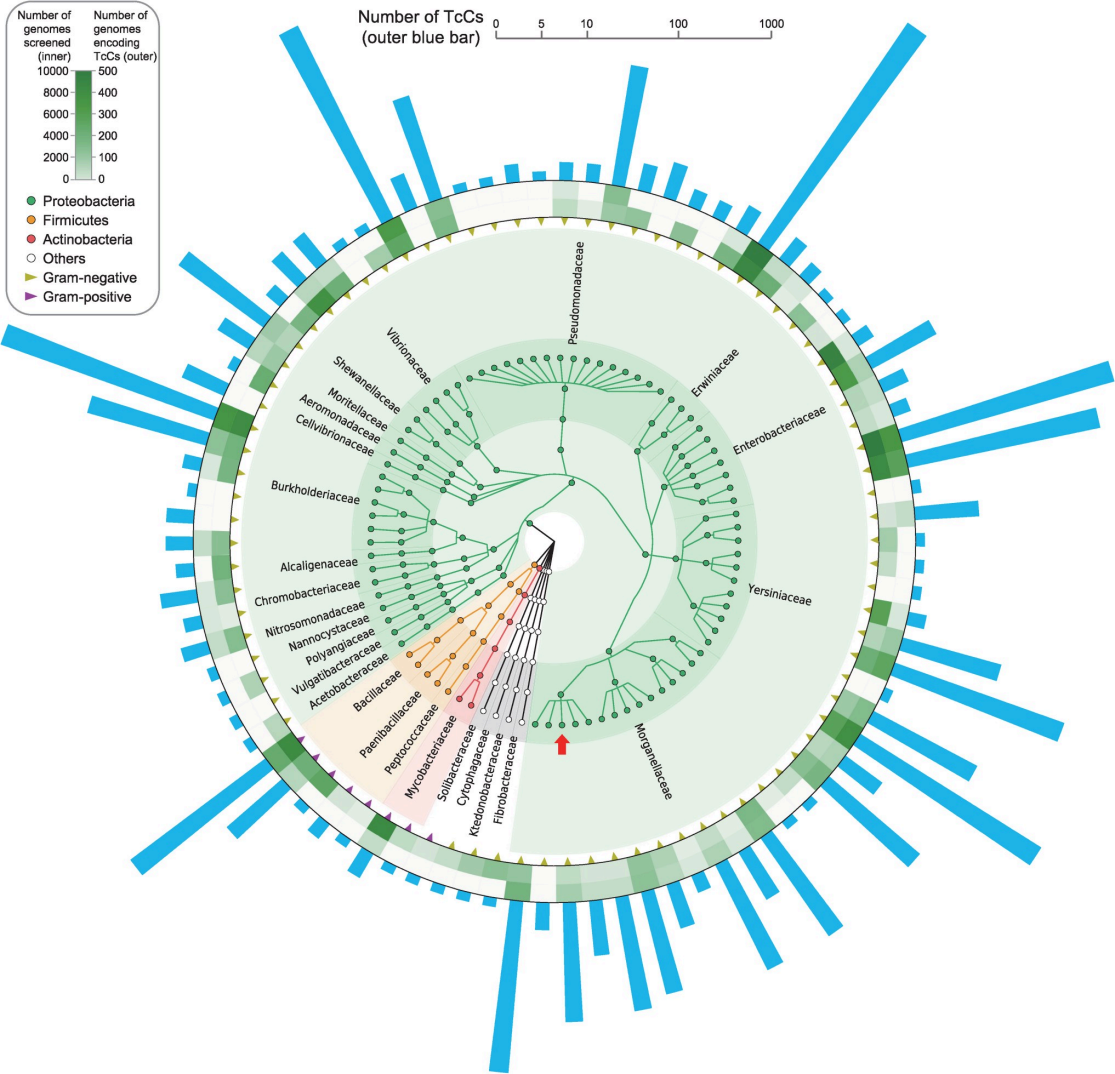
The Taxonomic Distribution of 2,528 Predicted TcC proteins among Bacteria. Only taxa with genomes encoding TcC proteins are shown for brevity. Seven genomes without known assigned family are excluded. The circles represent phylum, class, order, family, genus and species from inner to outer, and are color-coded by phylum (key). Genomes without a known species assignment are grouped into an individual circle within the corresponding genus. The family names are given outside the taxonomic tree. Triangles indicate species of Gram-negative (yellow) and Gram-positive (purple) bacteria. The heatmaps represent the total number of genomes screened (inner) and the number of TcC encoding genomes identified (outer) for each species (or unassigned group). The outer blue bars show the number of TcC proteins identified in a hybrid of linear (1–10) and log (>10) scale. The circle of *P*. *luminescens* species that includes the W14 strain (as shown in Fig 1) is indicated with red arrow.

Besides the 87 TcC proteins which are encoded within the genomes of Gram-positive bacteria, which are mainly from the phylum *Firmicutes* (3.2%), the majority of TcC proteins are found in Gram-negative bacteria, dominated by the phylum *Proteobacteria* (96.4%) (Fig 2). Nevertheless, the overall proportions of bacterial genomes that encode TcC proteins are about 1% and 3% for phyla *Firmicutes* and *Proteobacteria* respectively in our results, though the observed percentages are variable between bacterial families (S2A Fig). Amongst the *Proteobacteria*, as depicted in S2 Fig, TcC proteins are particularly prevalent in the class γ*-proteobacteria* (82.8%) and β*-proteobacteria* (13.3%), which include pathogens such as *Pseudomonas*, *Yersinia*, *Salmonella* and *Burkholderia*. This indicates that Tc toxins are widely distributed among bacteria, suggesting they could play an important role in the pathogenesis of infections. Notably, although over 13% of *Pseudomonas* genomes encode Tc toxins, they were predominant in *P*. *syringae*, *P*. *fluorescens* and many other species rather than *P*. *aeruginosa*.

Based on the findings described above, we have created an open-access database of all predicted Tc loci, named dbTC (http://www.mgc.ac.cn/dbTC/), to facilitate future experimental studies. This database provides an interactive linear map of each Tc locus with all Tc components color coded and highlighted with clickable link to gene details, along with meta-information, including taxonomy, genomic features, and any related publications, *etc*..

### The auto-proteolysis of TcC proteins

Experimental studies have shown that certain TcC proteins undergo an aspartyl auto-proteolysis process at the boundary between the conserved N-terminal region and the HVR, leading to the release of the TcC HVR domain into target cells as an independent (typically toxic) polypeptide [28,29,35]. Two aspartate residues, D651 and D674 in TccC3 of *P*. *luminescens* W14, were identified to be critical for auto-cleavage [35]. We constructed a *P*. *luminescens* W14 TcdB1-TccC2 fusion protein to investigate the role of residues upstream of the cleavage site, including PxxxxDxxG, which we showed to be well conserved among TcC proteins (S1C Fig).

**Fig 3:**
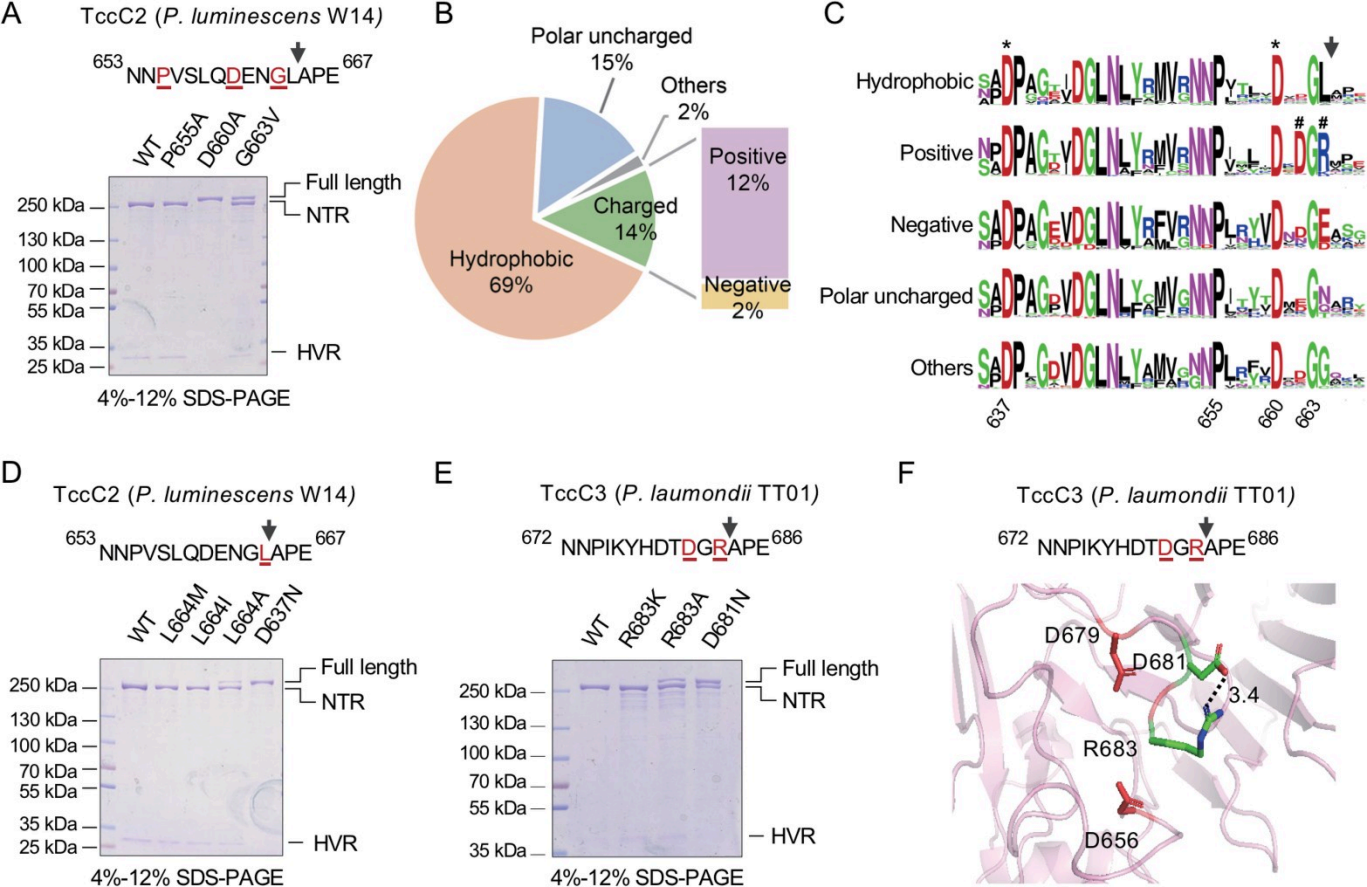
Auto-cleavage of the C-terminal HVR of TcC Proteins. (A) Cleavage of *P*. *luminescens* TcdB1*-*TccC2. The mutated amino acid residues were colored and underlined in the corresponding sequence (upper panel). The effect of the indicated mutations on auto-proteolysis was analyzed by SDS-PAGE. NTR, N-terminal region; HVR, C-terminal hypervariable region. (B) Pie chart showing the proportions of TcC proteins with different types of amino acid residues at the P1 position. (C) Comparison of sequence logos around the auto-cleavage site of indicated groups of TcC proteins. The black arrow indicates the auto-cleavage site. The residues that are required for the aspartyl protease activity are marked with *. The positive/negative amino acid pair is marked with #. The logos were constructed by WebLogo with default settings and re-numbered to match the residue numbering in TccC2 from *P*. *luminescens*. (D-E) Cleavage of *P*. *luminescens* TcdB1*-*TccC2 (D) and *P*. *laumondii* TcdB2*-*TccC3 (E). The mutated amino acid residues were colored and underlined in the corresponding sequence (upper panel). The effects of indicated mutations on auto-proteolysis were analyzed as shown in (A). (F) A structural model of the auto-cleavage active site of the *P*. *laumondii* TcdB2*-*TccC3 based on the cryo-EM structure of *P*. *luminescens* TcdA1-TcdB2-TccC3 holotoxin (Template: 6h6e.1.F, sequence identity: 91.37%, QMEAN: -1.14). The toxin is cleaved after R683. Two proteolytically essential aspartic acid residues are shown in red. The distance between the side chain of D681 and R683 is measured by PyMOL.

Consistent with previous studies [28,35], the mutations of D660 and G663, corresponding to D674 and G677 of *P*. *luminescens* TccC3, significantly abolished auto-cleavage (Fig 3A). In contrast, although the P655 residue (and its equivalent) is conserved in all identified TcC proteins, P655 mutation did not show any detectable effect on the auto-proteolysis (Fig 3A). Besides P655, several other conserved residues such as D643, N653, N654, were also not critical for the auto-proteolysis process (S3 Fig).

As the natural cleavage site is located between L678 (P-1) and M679 (P+1) in *P*. *luminescens* TccC3, we further analyzed the equivalent P-1 residue of the 2,528 TcC proteins we have identified. This allowed us to group them into five classes based on the chemical characteristics of this residue (Fig 3B and 3C). Sixty-nine percent of TcC proteins have a hydrophobic residue at the P-1 site. Substitution of this residue with other large hydrophobic amino acids, such as methionine, isoleucine, show no detectable effect on the auto-proteolysis, while L664A slightly affect the cleavage of TccC2, suggesting that hydrophobic residues with a large side-chain are preferred at the P-1 site for these aspartyl proteases (Fig 3D). It should be noted that, 29% of the P-1 site residues are either polar or charged in character. When the P-1 residue is positively charged (Arg, Lys and His), an aspartic acid residue is preferred in position P-3 (Fig 3C). To investigate whether this P-3 aspartic acid is required for the auto-cleavage of TcC subunits exhibiting a positively charged P-1 residue, we constructed a further BC fusion protein. This construct contains the TcdB2-TccC3 of *P*. *laumondii* TT01, the P-5 to P-1 sequence of which is Asp-Thr-Asp-Gly-Arg. Either of the D681N and R683A mutations, both of which alter the charge of the residues, were shown to negatively affect cleavage of the HVR. Conversely a R683K substitution, which maintains the positive charge of the P-1 residue, showed no adverse effect (Fig 3E). A homology model of *P*. *laumondii* TccC3 also suggested that a charge-charge interaction may occur between the side chains of D681 and R683, suggesting that the co-evolution of these two positions may be required for maintaining the autoproteolysis activity of TcCs (Fig 3F). Taken together, these observations not only confirmed the functional significance of the characteristic residues important for auto-proteolysis, but also implied that auto-cleavage is likely a common characteristic in many, if not all TcC proteins.

### TcC proteins are polymorphic toxins

**Fig 4:**
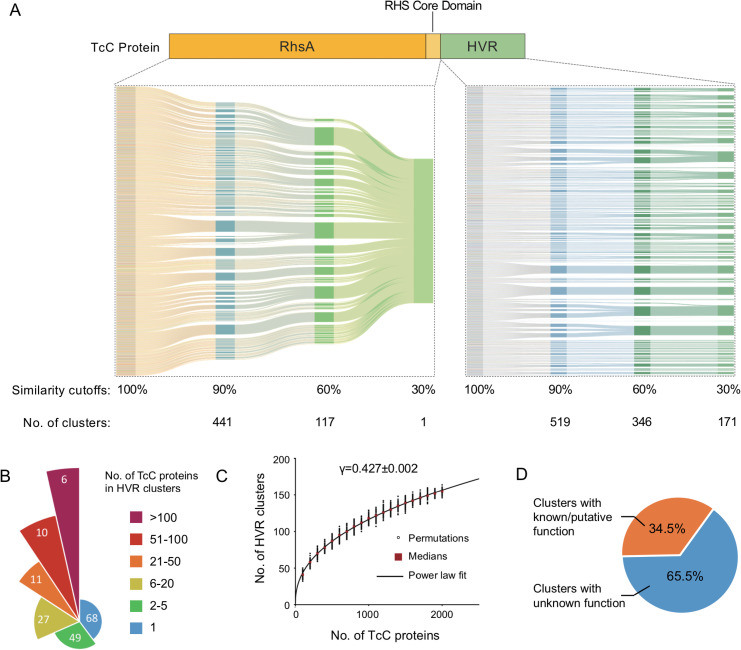
**TcC Proteins are Polymorphic Toxins with Variable HVRs** (A) The iterative clustering procedure of the N-terminal sequences of 2,528 TcC proteins. The sequences were clustered into 441, 117 and 1 cluster(s) at the similarity cutoffs of 90%, 60% and 30%, respectively (left). The iterative clustering procedure of the HVRs of 2,528 TcC proteins. The HVRs were clustered into 519, 346 and 171 clusters at the similarity cutoffs of 90%, 60% and 30%, respectively (right). The protein sequence clustering was conducted by CD-HIT. (B) A pie chart showing the uneven distribution of TcC proteins among the 171 HVR clusters. The central angle of each slice is proportional to the number of HVR clusters (inner number). The slice area is proportional to the total number of TcC proteins within all HVR clusters in each group. (C) Rarefaction simulation of the number of HVR clusters as a function of the number of TcC proteins sampled. Medians of 100 permutations (circles) for each value of TcC numbers are indicated by red squares. The solid line is a least-squares fit of the power law n = kN^γ^ to the medians. The exponent γ>0 indicates an open trend of HVR clusters. (D) Pie chart showing the proportions of HVR clusters with experimentally verified function or predicted domains (orange) and those without any homology to known protein domains (blue). Conserved protein domains were identified in the CDD database using the representative sequence of each HVR cluster.

As we confirmed, all TcC proteins have a conserved RHS core domain, which encodes a PxxxxDxxG motif (Figs 3C and S1C). To further dissect the genetic characteristic of TcC subunits, we used an iterative procedure to hierarchically cluster the N-terminal sequences (upstream of the cleavage site) of all 2,528 predicted TcC proteins (Fig 4A). At a 30% amino acid sequence similarity cutoff, the N-termini of these proteins form a single cluster. However, when applied to the variable TcC HVR regions, the same iterative clustering procedure classified them into 171 distinct clusters at 30% similarity cutoff (Fig 4A). These findings not only confirmed the reliability of our Tc identification pipeline but also demonstrated that TcC proteins do conform to the accepted definition of polymorphic toxins (PTs), exhibiting conserved N-terminal domains and C-terminal HVRs.

Interestingly, there is a striking imbalance in the number of TcC HVRs within each cluster. More specifically, 54 clusters (31.6%) consisting of >5 sequences account for approximately 91.6% of TcC HVRs in total, whereas 68 (39.8%) represent singleton HVR “clusters” (Fig 4B and S1 Table). Interestingly, the rarefaction curve of the numbers of distinct TcC HVRs versus the numbers of observed TcC proteins showed no sign of reaching a plateau. This implies that any new TcC proteins identified in future could well encode novel HVRs with no homologues in the current dataset (Fig 4C). Indeed, a Heap’s law fit of the curve gives a γ value of 0.427, which suggests the total number of distinct TcC HVRs is not yet predictable, and thus the extent of potential diversity remains an “open question”[36].

Considering only a few TcC HVRs have been experimentally confirmed to date, or even bioinformatically predicted encoding known toxin domains [22,31], we further examined the predicted protein domains in representative members of the different HVR clusters. Overall, among the 171 HVR clusters identified above, only 43 characterized domains could be ascribed predicted functions from 59 HVR clusters, whereas the majority (65.5%) of these clusters do not encode recognized domains (Fig 4D and S1 Table). Of the HVRs that did encode predicted functions, it was possible to recognize many well-studied domains typically encoded in other bacterial toxins or effector proteins (S1 Table). Examples include the Rho-activating domain of cytotoxic necrotizing factor CNF1, the actin-ADP-ribosylating toxin domain VIP2, heat-labile enterotoxin alpha chain Enterotoxin_a, and the cys-based protein tyrosine phosphatase domain (PTP_DSP_cys) [37,38]. This is indicative that “domain recycling” has occurred throughout the evolutionary history of the pathosphere’s gene pool, typical of polymorphic toxin systems.

Recent studies have suggested physical constraints for Tc toxins delivering cargo proteins, which require an isoelectric point (*p*I) higher than 8.0 [39,40]. To explore any common characteristics shared among potential cargo proteins, we further analyzed the biochemical properties of our panel of identified TcC HVRs. We note that 99.8% of the predicted TcC HVRs are smaller than 40 kDa, supporting the idea of an upper-limit for the capacity of the TcB-TcC cocoon (S4 Fig). We also noticed that, the *p*I values of natural HVRs are more variable than the previous studies imply regardless the taxonomic origins. For example, 36.5% of them, from various bacterial families, are lower than pH 7 (S4 Fig). To investigate whether the *p*I value is a stringent property for HVR release, we selected two natural HVRs, PTP of *Y*. *pestis* TcC (Accession: AKB86704.1, *p*I: 5.76) and CNF1 of *Y*. *entomophaga* TcC (Accession: ANI28957.1, *p*I: 7.95), and constructed two chimeric TcB-TcC fusion proteins, BC-PTP and BC-CNF1, which are composed of the N-terminal region of TcdB1-TccC2 and the indicated HVRs fused with a C-terminal Flag tag. Negative staining showed that purified TcdA1-TcdB1-TccC2 Tc toxin forms a pre-pore structure which closely resembles reported Tc toxins (S5A Fig). By using a cell-free in vitro translocation assay [39], we evaluated the release of cargo proteins in pH 8 and pH 11. As shown in S5B Fig, wild-type Tc particles assembled with TcdA1 and TcdB1-TccC2 do release the TccC2-HVR after 48h of incubation at pH 11. Similarly, both HVRs of BC-PTP and BC-CNF1 could also be released after incubation at pH 11 (S5C Fig). Although Tc particles formed by TcdA1 and BC-PTP did not show obvious effects on mammalian cells (S5D Fig), treatment of TcdA1-BC-CNF1 did lead to a change in cell morphology and apoptosis (S5E Fig). In contrast, a BC-CNF1-C818S mutant, which disrupts the catalytic activity of CNF1, did not affect the survival of the target cells, indicating that the pro-apoptotic effect is caused by the intracellular delivery of CNF1 HVR. Furthermore, we also fused RFP or GFP with the N-terminal region of TcdB1-TccC2 (S5F Fig). Similarly, RFP can still be released as a functional protein in the *in vitro* translocation assay (S5F and S5G Fig). This was also the case for GFP (*p*I: 5.67) (S5F and S5H Fig). Of note, the translocation of RFP in TcdB1-TccC2 cocoon works much better than that of GFP (pI = 5.67), probably due to the pI of TccC2 HVR is 8.7, suggested that in the TcB-TcC cocoon with a positively charged HVR, a positively charged cargo protein could be engineered. Taken together, our data suggested that, although the TcC HVR needs to be smaller than 40 kDa, the *p*I value does not appear to be critical for the HVR release, giving this system potentially broad biotechnology applications.

### The taxonomic distribution of TcC HVRs

**Fig 5:**
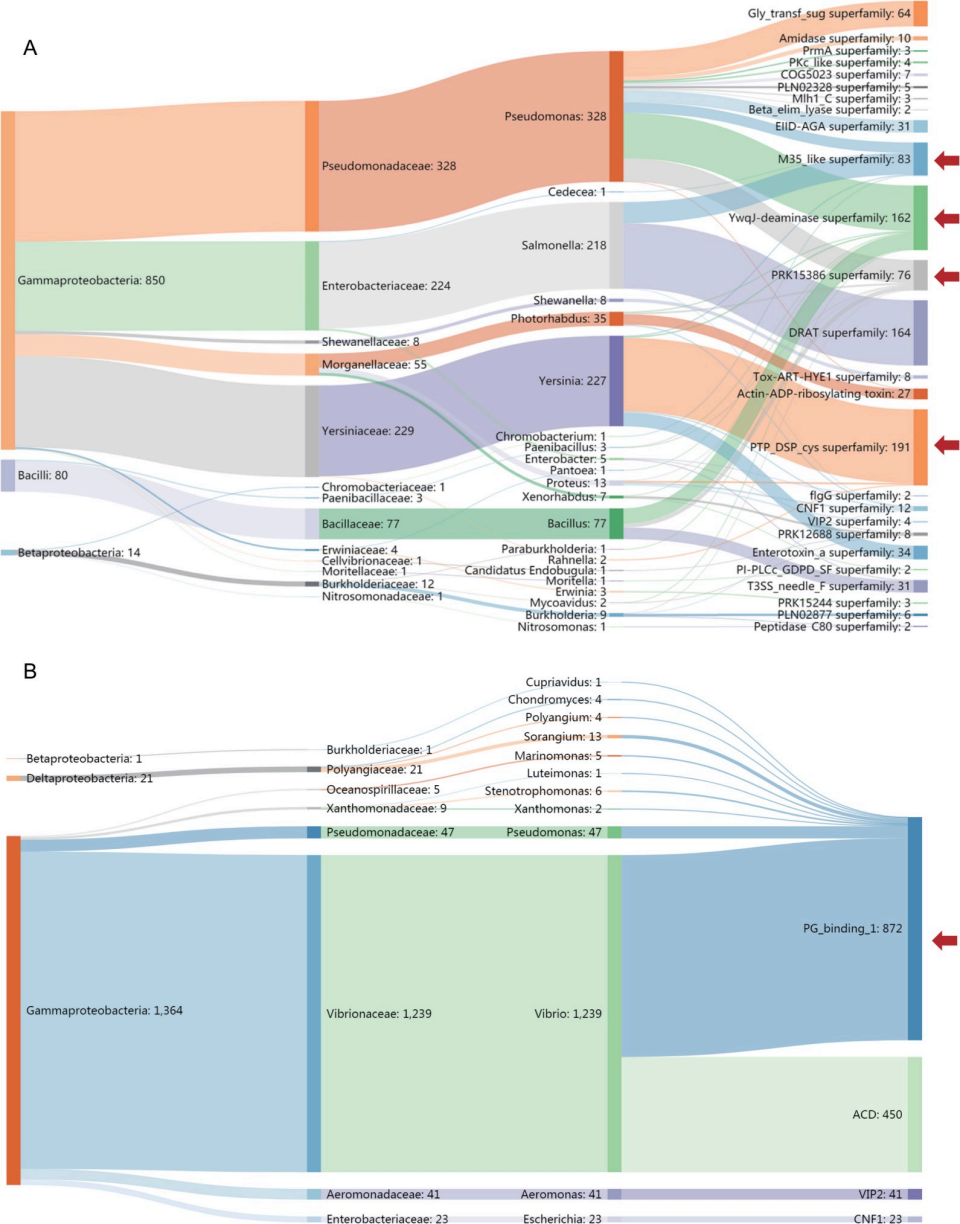
**The Taxonomic Distribution of TcC HVRs and Evolved VgrG C-terminal Regions** (A) Sankey diagram showing the relationship between bacterial taxa (class, family and genus from left to right) and the known or predicted function of HVR protein domains (rightmost). Domains present in only one HVR are excluded for clarity. (B) Sankey diagram showing the relationship between bacterial taxa (class, family and genus from left to right) and the known or predicted functions of evolved VgrGs (rightmost). The number of sequences involved in each node is given after the name of taxon or function/domain. The red arrows on the right indicate some typical examples of HVR domains that break the taxonomical specificity. The M35_like and YwqJ-deaminase domains both present in *Pseudomonas*, while they also present in *Salmonella* and *Bacillus*, respectively. Although PRK15386 and PTP_DSP_cys domains are predominant in *Pseudomonas* and *Yersinia* respectively, but there are also a few exceptions (slim curves) that present in other genera.

As discussed above, the TcC proteins are widely distributed among different bacterial genera and contain a variety of HVRs as effector domains. We performed a Sankey analysis to investigate the relationship between the types of HVR and the bacterial genera which encode them. Interestingly, we found that most of the HVR clusters encoding characterized domains showed an obvious genera specific distribution with limited exceptions, correlating well with the originating taxa of TcC proteins (Fig 5A). Of note, this distribution pattern differs from that of effector domains found in other secretion systems, which are typically shared by a variety of bacterial species. For instance, the VIP2 domain exhibits an ADP-ribosyl transferase activity in many T3SS effectors, such as ExoS in *Pseudomonas*, VopT in *Vibrio*, SpvB in *Salmonella*, and YspE in *Yersinia* [41–44]. In contrast, amongst the 2,528 predicted TcC proteins in our dataset, TcC proteins contain VIP2 domain are only found in the genus *Proteus*. Similar examples in well-characterized T3SS effectors include the PKc_like catalytic domain of protein kinase superfamily that present in OspG (*Shigella*), YpkA (*Yersinia*), Pkn5 (*Chlamydia*) and AopO (*Aeromonas*), and the SseC domain of secretion system effector C like family that present in SseC (*Salmonella*), IpaB (*Shigella*), CopB2 (*Chlamydia*) and BipB (*Burkholderia*), since TcC proteins encoding PKc_like and SseC domain are limited in the genus *Pseudomonas* and *Nitrosomonas* respectively in current result (S1 Table).

Considering the majority of TcC HVR clusters exhibit a genera specific distribution, we decided to examine whether other polymorphic toxins also share this characteristic. As T6SS effectors, the evolved VgrG proteins are defined as typical PTs members, with variable C-terminal domains, some of which have been experimentally demonstrated (or predicted) to encode conserved toxic domains [15,16]. It is interesting to note that 41 publicly available evolved VgrGs containing VIP2 domain are all derived from the genus *Aeromonas*. Moreover, all of the 450 evolved VgrG proteins available from GenBank, which carry the actin cross-linking domain (ACD), are exclusively *Vibrio* proteins. Furthermore the 872 evolved VgrG proteins encoding the peptidoglycan binding domain (PG_binding_1) are predominantly derived from *Vibrio* (90.5%) and *Pseudomonas* (5.4%), with very few exceptions (Fig 5B). The taxonomic distribution of conserved C-terminal domains previously determined in evolved VgrGs does reveal a similar pattern to that observed for the TcC HVRs.

### TcC proteins are widely distributed among *Salmonella* and *Yersinia*

To further explore the presence of Tc toxins in finer detail, we extended our search to screen multiple draft *Salmonella* and *Yersinia* genomes available in EnteroBase, which hosts the largest worldwide collection of genomes from these two genera [45]. With the same criteria as detailed above, our scans discovered 7,024 TcC proteins in 2,995 *Salmonella* genomes, and 1,940 TcC proteins in 1,693 *Yersinia* genomes, respectively.

**Fig 6:**
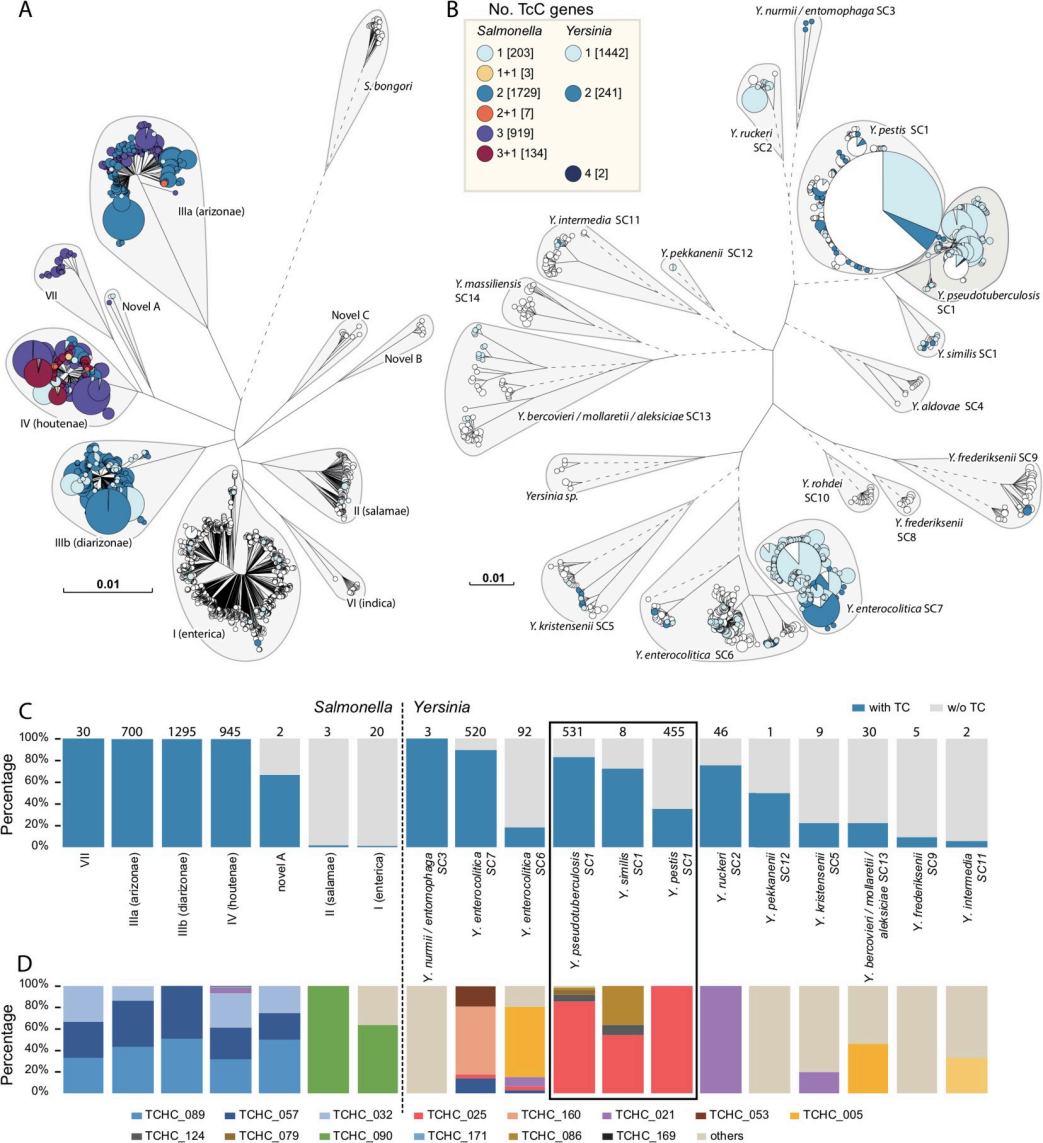
**Distribution of TcC Proteins in *Salmonella* and *Yersinia* Present in EnteroBase** (A-B) Neighbor-joining phylogenies based on concatenated sequences of 5,314 selected *Salmonella* genomes (A) or all 3,341 *Yersinia* genomes (B) in EnteroBase. Phylogenies were visualized using GrapeTree and branches with a genetic distance ≤ 1e-6 in both trees have been collapsed for clarity. The size of each circles is proportional to the number of genomes that are assigned to the node. The pie charts in each circle were color-coded by the number of *tcC* genes in each genome, as shown in the Key. All *Salmonella* genomes (A) were further separated into subspecies, and the *Yersinia* genomes (B) were separated into species complex, with the exception of SC1 which is further separated into the three recognized species. Taxonomic groups are shown with grey shadowing. (C) The percentages of genomes that encode TcC proteins (blue) or do not (grey) for each of the taxonomic groups. The total number of TcC encoding genomes within each group is listed above column. (D) The percentages of HVR clusters in the Tc encoding genomes in each taxonomic group. Only the 14 most common HVR clusters (n>10) are shown for visual clarity.

To analyze the phylogenetic distribution of Tc toxins in *Salmonella* and *Yersinia*, a set of 5,314 *Salmonella* and 3,341 *Yersinia* genomes that represent the entire known genetic diversity of these two genera was collected to calculate a neighbor-joining tree for each genus. Over 90% of the Tc loci in *Salmonella* carry two or three copies of *tcC* genes (Fig 6A). In contrast, over 85% of the Tc loci in *Yersinia* carry only a single copy of a *tcC* gene (Fig 6B).

It is interesting to note, the distribution of TcC proteins among (sub-) species of *Salmonella* and *Yersinia* is uneven (Fig 6C). The presence of Tc toxins in *Salmonella* are largely restricted to *S*. *enterica* subspecies IIIa, IIIb, IV, VII and novel subspecies A. Almost all genomes in these subspecies carry at least one Tc locus. In contrast, after the assignment of each of the *Yersinia* genomes into one of the 15 species complexes (SCs) as previously defined [46], Tc toxins are encoded in at least 10 of these SCs and with varied proportions.

Using the aforementioned clustering procedure, together with the TcC HVRs from the GenBank dataset, a total of 36 distinct clusters of HVRs were identified in the *Salmonella* and *Yersinia* datasets, 10 of which are different from the above 171 clusters identified in the RefSeq dataset (S2 Table). The 10 new HVRs account for only 1% of the TcC proteins in the two genera despite the massive number of genomes deposited in EnteroBase. This finding also confirms the rarefaction curve drawn from the RefSeq dataset, and further reveals the genetic complexity of HVRs in nature.

Most of the TcC HVRs are exclusively encoded in *Salmonella* or *Yersinia*, with the exception of TCHC_021, TCHC_038, and TCHC_057, which are present in both. The *Yersinia* genomes encode 27 distinct clusters of TcC HVRs. Despite that nearly three-fold more *tcC* genes were found in *Salmonella* than in *Yersinia*, even though the *Salmonella* TcC proteins show less genetic diversity and are only separated into 12 distinct clusters. As Fig 6D indicates, most of the TcC HVRs found in *Salmonella* subspecies (IIIa, IIIb, IV and VII) fell into one of the three clusters: TCHC_089, TCHC_057, and TCHC_032. These TcC HVRs have not been found in other *Salmonella* subspecies. Only very few TcC proteins have been found in *Salmonella* subspecies I and II, most of which carry HVRs of TCHC_090 domain (Fig 6D). In contrast, each *Yersinia* SCs mainly have a different set of TcC HVRs, with the exception that several TcC HVRs, such as TCHC_025, TCHC_021 and TCHC_005, were found in multiple species. Our results indicate that, although Tc toxins are widely encoded in both *Salmonella* and *Yersinia*, these two genera exhibit quite different distribution patterns of Tc toxins.

### A two-level evolutionary scenario of Tc toxins in *Salmonella* and *Yersinia*

**Fig 7:**
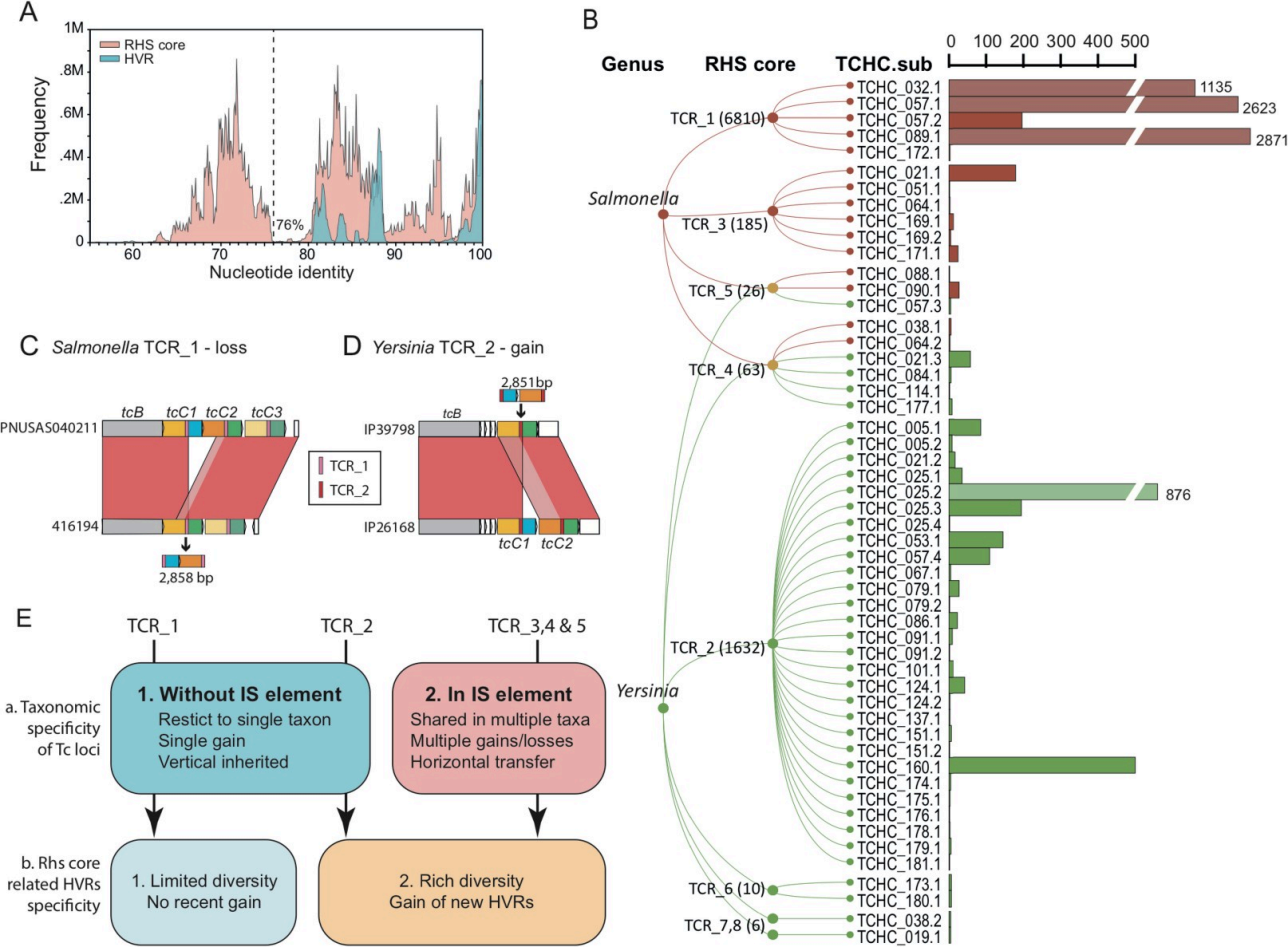
**The Evolutionary Dynamics of TcC Proteins in *Salmonella* and *Yersinia*** (A) Histogram of the all-against-all BLASTn comparisons of the nucleotides encoding RHS core domains (pink) and the HVRs (blue) for all identified *tcC* genes. The BLASTn results are binned every 0.5%. A dotted line shows the cutoff level (76%) that was used to separate both regions into their respective groups. (B) Left: A cladogram that shows the correlation between the RHS core groups and the HVR sub-clusters of the two genera. Right: A bar-chart of the numbers of the genomes that encode each of the HVR sub-clusters in EnteroBase. Genus is color-coded by red (*Salmonella*) or green (*Yersinia*). RHS core domains were divided into 8 distinct groups (namely TCR_1 to TCR_8). The number of TcC proteins in each TCR group are shown in the brackets. The aforementioned 36 HVRs clusters were further split into 52 sub-clusters (*e*.*g*. TCHC_025.2). Each HVR sub-cluster is uniquely associated with only one RHS core group, whereas one RHS core group can be associated with up to 28 HVR sub-clusters. (C-D) Exemplar of the apparent gain/loss of a 2.8 kb fragment in the Tc loci between two genetically closely related *Salmonella* (C) or *Yersinia* (D) genomes. Homologous regions between two genomes are connected by red (high identity) or pink (low) parallelograms. The domains that are gained or lost are illustrated as a putative episomal fragment, surrounded by sequences encoding RHS core domains (color-coded as in Key). (E) A hypothetical two-level model for the evolution of TcC proteins in *Salmonella* and *Yersinia*. Tc locus that encode each RHS core group differs from other Tc loci by its evolutionary pattern of either vertical inheritance or horizontal gene transfer. Variation of TcC protein in each Tc locus is regulated by the diversity of possible HVRs for each RHS core group.

As TcC proteins and their HVRs exhibit different distribution patterns in *Salmonella* and *Yersinia* (Fig 6), we investigated the evolutionary dynamics of the Tc toxins in these two genera in greater detail. The pairwise comparisons of all the RHS core and the HVR sequences from *Salmonella* and *Yersinia* show similar genetic discontinuity patterns (Fig 7A). Only ~0.2% of pairwise comparisons of RHS core sequences fell between 76% and 79% nucleotide identities, and <0.1% of pairwise comparisons of HVR sequences fell between 50% and 80% nucleotide identities. Therefore, to reflect the genetic discontinuum of both domains, we applied average-linkage clustering with a cutoff at 76% nucleotide identity to divide TcC RHS core domains in *Salmonella* and *Yersinia* genomes into 8 distinct groups (namely TCR_1 to 8) and divided HVRs into 52 distinct sub-clusters (Fig 7B).

Except in three *Yersinia* species complex SC3 genomes which carry TcCs that encode TCR_7 and 8 within the same Tc locus, all other Tc loci with multiple TcC copies encode single TCR group (S6A and S6B Fig). Tc loci encoding TCR_1 and 2 are exclusively predominant in *Salmonella* (2,924 genomes from 5 subspecies) and *Yersinia* (1,632 genomes from 6 species complexes), respectively (Fig 7B). Furthermore, we analyzed the genomic neighborhood of the different TCR loci, and found that TCR_1 encoding loci always locate between the same two housekeeping genes (*yejM* and *narP*) in *Salmonella*, while TCR_2 encoding loci are found between homologous of YPO3672 and YPO3682 in *Yersinia* (S6C Fig). A maximum parsimony inference of the evolution of these two TCR groups suggests that each was present in a common ancestor of the genus and vertically passed to their descendants, which allows Tc loci to be fixed into these two bacterial genera (S7A Fig).

Furthermore, 6% of Tc loci encoding three minor TCR groups, TCR_3–5, are all sandwiched by transposases, and have highly homoplastic phylogenies (S6C Fig). Indeed, TCR_4 and 5 encoding loci are present in both *Salmonella* and *Yersinia* (Fig 7B). In addition, all these three TCR encoding loci have been identified from *S*. *enterica* subspecies I, which is an important human pathogen and appears to have lost its ancestral Tc locus (TCR_1) before emergence (S6A Fig). These Tc loci which seem to be carried by mobile elements could allow horizontal transfer into taxonomic groups that have no ancestral Tc locus, and possibly be responsible for the low-level Tc carriage observed in many other bacterial genera (S7A Fig).

As the N-termini and HVR regions of *tcC* genes are evolutionary dissociated in *Salmonella* and *Yersinia* (Fig 7A), we compared two genetically related yet diverged Tc loci which carry different copy numbers of the *tcC* gene. We observed an additional 2.8-kb fragment containing the HVR of *tcC*1 gene and RhsA domain of the consecutive *tcC*2 gene in the relevant Tc locus (Fig 7C and 7D). Notably, this fragment is surrounded by the direct repeat of the RHS core domain. Previous studies indicated that the efficiency of homologous recombination decreases rapidly with increasing sequence divergence [47]. As our findings show that each HVR sub-cluster was only associated with single TCR group, we propose a mechanism for the replacement of TcC HVRs. In this scenario, a small episomal DNA circle can be gained or lost by homologous recombination in the RHS core repeat belonging to the same TCR group. This would thus cause not only the replacement of the downstream HVR but also allow for copy number variation of *tcC* genes within the locus (S7B Fig).

Therefore, we propose a “two-level” model to explain the genera-specific distribution of Tc toxins, in which Tc loci are vertically inherited into their descendants as the main drive combined with limited horizontal transfers mediated by mobile elements. Sequence similarity of the different conserved RHS core domains further regulates the gain/loss of specific TcC HVRs (Fig 7E). Our Sankey analysis did show the taxonomical specificity of TcC HVRs, with only a few exceptions distributed cross different genera (Fig 5A).

In accordance with this model, we explored the divergent Tc toxin distribution among *Salmonella* and *Yersinia*. The *tcC* genes in *Salmonella* appear to be evolutionarily “stable”, since only three intact HVRs (TCHC_032.1, TCHC_057.1 and TCHC_089.1) or their truncated versions (TCHC_057.2 and TCHC_172.1) have been found in all 2,927 TCR_1 encoding loci. The order of these *tcC* genes are highly conserved without any observable rearrangement, duplication or insertion. However, the TCR_2 encoding Tc loci in *Yersinia* appear to have been actively acquiring new HVRs, which implies diversifying selection. For example, TCHC_025.3 is present in *Y*. *pestis* but not in its direct ancestor *Y*. *pseudotuberculosis*. These results indicate the TcC protein distribution in *Salmonella* and *Yersinia* have different evolutionary dynamics, in which *Salmonella* carry about three-fold more TcC proteins while encoding less than half HVR clusters found in *Yersinia*, as shown in Fig 6C and 6D.

## Discussion

Here, we present a protein-profile and genomic context-based approach to identify potential Tc toxins in publicly available bacterial genomes. A total of 2,528 TcC proteins were predicted within 1,608 genomic loci, which distribute among 1,421 genomes of Gram-negative/positive bacteria. Further bioinformatic and experimental approaches were performed to explore the common characteristics of this important polymorphic toxin family.

Although Tc toxins are recognized as entomopathogenic toxins in many insect-related bacteria, our results show that homologues of Tc toxin are also encoded in many human pathogenic bacteria such as *Salmonella* and *Yersinia*. This implies that this toxin delivery apparatus is likely to play more important roles in the lifecycles of more diverse bacteria than previously expected (Figs 2 and 6). Even so, we believed that the prevalence of Tc toxins we have identified is likely an underestimate for the following reasons. Firstly, the distribution of TcC proteins in other phyla of bacteria might be underestimated due to the sampling bias in current bacterial genome sequencing projects, of which >50% are Proteobacteria. Secondly, as *tcC* genes encode RHS core domains, which represent repetitive elements, they might lead to fragmentation during the assembly of draft genomes. Since nearly 90% of the genomes analyzed here are draft genomes, we would not have been able to “reconstruct” any such fragmented Tc loci given our strict screening criteria. Thirdly, to exclude potential contamination of the database with Rhs proteins, which also carry the same RHS core domains as TcC proteins, we set the screening criteria to only include TcC subunits which are encoded adjacent to *tcA* or *tcB* gene homologues. However, manual examination of several bacterial genomes has shown that there are indeed cases of ‘orphan’ *tcC* homologues encoded independently of *tcA* or *tcB* genes in the immediate genomic vicinity. For example, TccC6 of *P*. *laumondii* TT01 is a typical TcC protein with C-terminal YwqJ-like deaminase domain. Based on information gleaned from our analysis of the TcC proteins identified here, a set of additional stringent criteria was devised to further screen for so-called “orphan TcCs” in complete bacterial genome sequences. This approach identified an additional 234 putative orphan TcCs, which have been included in the database as an independent category. As it remains formally possible that the proteins encoded by these homologues could be delivered by means other than the well-defined Tc-injectosome mechanism, further biological investigations are required before we ascribe this subset as *bona fide* Tc delivered effectors. In addition, there are also several examples of *tcB* and *tcC* fusion genes encoded in some bacterial genomes, *i*.*e*. a single gene encodes an apparent B–C fusion protein (Accession: ABA52082.1) in *B*. *pseudomallei* strain 1710b. Although these samples are not included in this study for clarity, it indicated that Tc toxins represent a more complicated system than previous expected.

Many previously described PTs are used for inter-bacterial competition, as such they typically encode immunity proteins for self-protection [10]. However, no predicted immunity proteins have yet been identified in any of the Tc loci we have examined. This is consistent with the current understanding that Tc toxins have evolved to target eukaryotic cells rather than bacteria [17,18,24,26,31]. It is of note that 65.5% of the 171 distinct HVR clusters contain no recognizable domains (Fig 4D). It is reasonable to hypothesize that these clusters utilize as yet unknown mechanisms for Tc-enabled pathogenesis. On the other hand, it is also interesting that many clusters identified here do encode known toxin domains, which are employed not only in other PTs but also in well-established bacterial toxins and secretion system effectors (Fig 5A and S1 Table). Taken together, these observations show that while there is a large shared pool of recognized toxin domains used by different virulence systems, there still remains many others which are as yet uncharacterized. Our higher resolution analysis of the TcC HVRs in *Salmonella* and *Yersinia* genomes using EnteroBase, revealed a large number of domains of unknown function (S2 Table). This is correlated with the rarefaction curve analysis, implying yet more distinct HVRs would be expected to be discovered with more available genomes (Fig 4C).

Previous studies have suggested a unified C-terminal pool for *rhs* genes across the *Enterobacteriaceae*, with a C-terminal replacement driven by recombination between RHS core domain sequences [48]. Our analysis suggested a similar RHS core dependent, homologous recombination driven replacement of TcC HVRs in *Salmonella* and *Yersinia*. Such a recombination, however, has been largely restricted to a single genus, due to a process we summarize as a “two-level” model. We propose that similar evolutionary dynamics may also occur in other PTs systems encoding a conserved N-terminal region. It is worth noting though, our two-level model reveals possible evolutionary histories of Tc loci in *Salmonella* and *Yersinia* that lead to their current, distinct TcC HVR distributions, but leaves open the cause of such differences. For example, the Tc loci in *Yersinia* are evolutionarily “active”, and has gained many new TcC HVRs, leading to a highly diverse TcC HVR pool, while the TCR_1 associated Tc loci in *Salmonella* are “stable”, and have not gained any new TcC HVRs after their last common ancestor. We speculate such differences may result from their adaptation to different hosts. Many *Yersinia* species, including important human pathogens *Y*. *pestis* and *Y*. *pseudotuberculosis*, are also adapted to infect insects, and Tc toxins are known to play a role in such a process [23,26,49]. In contrast, the main niche of *Salmonella* is the intestinal tract of mammals, wild birds, reptiles, but occasionally insects [50]. Therefore, the Tc toxins encoded by the common ancestor of *S*. *enterica* might help the bacteria living in insects, but have become less useful later and therefore lost repetitively in multiple subspecies. Additional investigations, however, are needed to support this speculation, because some *Yersinia* species that have not been associated with insects, such as Y. *enterocolitica* which is a food-borne pathogen primarily found in mammals [51], and *Y*. *ruckeri* which is mostly isolated from fish [52], still encode Tc toxins.

The open access database we present here will also facilitate further studies of this currently underestimated toxin superfamily, not only for the study of virulence mechanism, but also for potential therapeutic and biotechnological applications.

## Materials and methods

### Plasmid

The coding sequences of TcdA1 (AAL18486.1), TcdB1 (AAL18487.1), TccC2 (AAL18492.2) were PCR cloned from the genome of *Photorhabdus luminescens* W14. TcdB2 (CAE13264.1) and TccC3 (CAE13262.1) were cloned from the genome of *P*. *laumondii* TT01. The coding sequences of CNF1-HVR of TcC (ANI28957.1) of *Yersinia entomophaga* and PTP-HVR of TccC1 (AKB86704.1) of *Yersinia pestis KIM10+* were synthesized by Ruibio BioTech. The coding sequences of GFP and RFP were cloned from pCMV6 vectors (PS100019 and PS100033, Origene). TcdA1 of *Photorhabdus luminescens* W14 was cloned into RSFDuet-1 (Novagen). All TcB-TcC fusion proteins were constructed incorporating a 5-aa linker region encoding Arg-Gly-Ser-Arg-Pro, and cloned into pETDeut-1 (Novagen) with N-terminal 6xHis tag. An AscI digestion site was introduced into the TcdB1-TccC2 fusion gene derived from *P*. *luminescens* W14 for the generation of chimeric TcB-TcC proteins including BC-PTP, BC-CNF1, BC-GFP, BC-RFP. Indicated HVR coding sequences were inserted into the pETDeut-1-TcdB1-TccC2 digested by AscI and NotI, respectively. To engineer the mutants of CNF1, *P*. *luminescens* TccC2, and *P*. *laumondii* TccC3, site-directed mutagenesis was performed according to the manufacturer’s protocol (TransGen Biotech). All constructs were confirmed by DNA sequencing (Sangon Biotech, Shanghai).

### Protein purification

Tc toxin-related proteins were prepared according to the previous protocol with some modifications [29–31]. All TcdA proteins were expressed in *E*. *coli* BL21 (DE3) cells (TransGen Biotech). The cells were collected and sonicated. Cell lysates were applied to a Ni-NTA affinity column (Qiangen), and the final eluate further purified using gel filtration (Superose 6 Increase 10/300 GL GE Healthcare) using an AKTA avant25. The TcB-TcC expressing plasmids were transduced into BL21-CodonPlus (DE3)-RIPL cells (Agilent Technologies). Expression was performed in 1L LB medium with 25 μM IPTG induction for 4 h at 28°C, with aeration by 250 rpm shaking. This was followed by 20 h at 24°C and a further 24 h at 20°C. The cells were harvested by centrifugation and lysed by sonication. Target proteins were purified by anion exchange chromatography, followed by a Ni-NTA affinity column and final gel filtration as above. Samples were subjected to 4%-12% SDS-PAGE separation. Both TcA and TcB-TcC fusion proteins were concentrated to ~1 mg/ml, using Amicon filter devices (Millipore).

For the purification of the holotoxin complex formed by *P*. *luminescens* TcdA1 and TcdB1-TccC2 fusion proteins, TcdA1 and TcdB1-TccC2 were mixed in a 1:1 mole ratio with a buffer containing 50 mM Tris pH 8.0, 100 mM NaCl, and 5% glycerol. The mixture was then applied to gel filtration using a Superose-6 10/300 GL column. The elution fractions between 12–14 ml were concentrated and finally used for negative staining or cell intoxication assay.

### Negative stain electron microscopy

After gel filtration, 4 μl TcdA1-TcdB1-TccC2 holotoxin sample droplets were applied on freshly glow-discharged copper grids (Agar scientific; G2400C) covered by a thin, continuous carbon film. The samples were left for 20 s on the grid before blotting with filter paper, and stained with 0.04% uranyl acetate twice, air-dried for 20s. All images were taken with a JEOL JEM-1400 electron microscope equipped with a LaB6 cathode operated at 120 kV. Digital electron micrographs were recorded with a 4k x 4k CMOS camera F416 (TVIPS) using minimal dose conditions.

### Cell intoxication

HEK293T or HeLa cells were seeded into 24-well plate (1 × 10^5^ per well), and grown adherently on sterile coverslips overnight in 500 μL culture medium. The indicated holotoxin was subsequently added. All the images were captured by EVOS FL Auto 2 Imaging System (Thermo Fisher Scientific).

### *In vitro* protein translocation assay

Purified holotoxin complex was mixed with n-Dodecyl β-D-maltoside (DDM) and dialyzed against Tris buffer (20 mM Tris-HCl pH 8.0, 150 mM NaCl, 0.1% DDM) or CAPS buffer (20 mM CAPS pH 11, 150 mM NaCl, 0.1% DDM) for 24 h at 4°C. Then the dialyzed samples were applied to gel filtration by using Superose-6 10/300 GL column. Each fraction was collected and analyzed by Western blot. In brief, samples were subjected to 12% SDS-PAGE and then transferred onto PVDF membranes. Membranes was incubated with indicated primary antibodies and corresponding secondary HRP antibodies, and examined by using enhanced chemoluminescent detection reagent (Thermo Fisher Scientific). The fluorescence of GFP or RFP was determined using a Cytation 5 Cell Imaging Multi-Mode Reader (BioTek Instruments, Winooski, VT). The red fluorescence cube is configured with a 542/20 nm excitation filter, a 594/20 nm emission filter, while the green fluorescence cube is configured with a 475/20 excitation filter, a 545/20 emission filter.

### Generation of the homology models

The generation of the homology structural models was performed using SwissModel. For *P*. *laumondii* TcdB2*-*TccC3, the cryo-EM structure of *P*. *luminescens* TcdA1-TcdB2-TccC3 holotoxin (PDB: 6h6e.1.F) was used as a template. The distance between D681 and R683 were calculated using PyMOL.

### Identification of Tc candidates among bacterial genomes

The previously characterised Tc loci from *Photorhabdus luminescens* W14 [17], *Enterobacter* sp. 532 [21] and *Serratia entomophila* A1MO2 [53] were manually collected from GenBank as exemplars for the genetic composition and organisation of Tc locus (accessions: AF346500, LC381419 and AF135182, respectively). A prototypical Tc locus usually encodes three proteins: TcA, TcB and TcC (Fig 1A). Conserved protein domains among Tc proteins were identified by the CD-search service of the Conserved Domain Database (CDD) with default parameters [54] (accessed at Jan, 2019). The VRP1 and SpvB domains are specifically related to proteins encoded on the Salmonella virulence plasmid rather than Tc proteins. So they were considered unsuitable for the current study. The Rhs associated core domain (RHS core domain) is the most conserved part of the RhsA domain. Therefore, in order to maximize the specificity and sensitivity of further screening, known domains TcdB_toxin_midN/TcdB_toxin_midC (PF12256/PF12255) and RHS core domain (TIGR03696) were selected as indicators of homologous of TcB and TcC, respectively (Fig 1A). The HMM profiles of the selected domains were retrieved from related databases for further analysis. Since no Tc-specific domains have been identified in TcA proteins thus far, the protein sequence of TcA from *P*. *luminescens* W14 (accession: AAL18486) was used as the initial query of the position specific iterative BLAST search in the non-redundant protein database of GenBank with an E-value threshold of 0.1. After five iterations of search the top 500 hits of the results were collected to construct multiple sequences alignment (MSA) using the Clustal-Omega program v1.2.4 [55]. Then, the MSA file was used to build HMM profile of TcA by the hidden Markov models as implemented in the HMMER3 package v3.1b2 [56].

All complete or draft prokaryotic genomes available from RefSeq (accessed at March, 2019) were downloaded to local system and parsed with BioPerl [57]. Genomes without annotations were excluded from further analysis, which yielded a set of 133,722 valid genomes, including 131,756 Bacteria and 1,966 Archaea. To systematically identify putative TcC proteins among bacterial genomes the aforementioned collected and constructed HMM profiles were used as seeds for a modified protein profile and genomic context-based pipeline previously proposed [58]. Since both RhsA and RHS core domain are also highly conserved in the Rhs proteins themselves, to avoid swamping the dataset with Rhs genes an empirical criterion was applied to the screening pipeline which include only TcC-like proteins encoded within the same genomic neighbourhood (<10 kb) to TcA or TcB genes. Moreover, an additional criterion we applied was that valid TcC proteins should have relatively complete RhsA domain (>400 aa), which is responsible for binding it to the TcB subunit. The same strategy was also applied on 374,773 bacterial genomes from seven genera deposited in EnteroBase (accessed at October, 2019) [45]. We found 1,940 TcC proteins encoded by 1,693 *Yersinia* genomes and 7,024 TcC proteins encoded by 2,995 *Salmonella* genomes, and no TcC protein in any of *Escherichia*, *Helicobacter*, *Clostridioides*, *Vibrio* and *Moraxella* databases.

### Phylogenetic and taxonomic analysis of TcC proteins

Due to the intrinsic logic of our screen strategy the RHS core domain is present in all TcC candidates, serving as the best molecular marker for phylogenetic analysis. The Clustal-Omega program was used to generate MSA of the RHS core domain region of all TcC candidates. FastTree program v2.1 was then employed to construct the maximum-likelihood phylogenetic tree under WAG models with gamma optimization [59]. Additionally, a 16S rRNA-based phylogenetic tree of life was available from the All-Species Living Tree project [60]. Only clades related to the phylum Proteobacteria were included for further visualization of the tree.

The iTOL [61] and GrapeTree [62] online servers were used to manipulate and visualize the different phylogenetic trees. The taxonomic distribution of TcCs were presented by GraPhlAn program [63]. The sequence logos of RHS core domain of various groups were generated by the WebLogo software v2.8.2 based on the MSA file [64].

### Hierarchical clustering of N- or C-termini of TcC proteins

TcC proteins are members of PTs family, which has bipartite architecture with a conserved N-terminal region fused to a variable C-terminal domain. Therefore, based on the location of RHS core domain each identified TcC protein was divided into two parts, N- and C-termini respectively. In an attempt to effectively cluster the N- or C-termini of TcC proteins at very low threshold (i.e. 30% amino acid identity), a hierarchical method with incremental neighbor-joining algorithm as implemented in the CD-HIT package v4.6.5 was employed [65]. Specifically, the protein sequences were firstly clustered with k-mer size of 5 at the thresholds global identity of 90% and alignment coverage of 80%. Then, the representative sequence of each cluster produced from the previous step were re-clustered with loosened settings of both *k*-mer size (*k* = 4) and global identity (60%). Finally, the cutoff was further decreased to allow clustering of former representative sequences with either global identity of 30% or BLAST E-value of 1e-6. The hierarchical clustering procedure was performed on protein sequences of N- or C-termini of all TcC proteins separately. Additional HVRs of TcC proteins identified in the EnteroBase database were clustered along with the former established HVR clusters by the same procedure. Particularly, 29 *Salmonella* TcC proteins with HVRs missing or truncated (< 10 aa) were excluded from clustering analysis.

### Experimental identification of potential orphan TcC proteins

In order to exclude the contamination of diverse Rhs element genes in our dataset, we used the criteria of proximity to TcA/TcB genes as an additional requirement for the identification of valid TcC in previous analysis. However, this also led to the absent of some known “orphan” TcC proteins from our dataset, such as TcC6 and TcC7 from *P*. *luminescens*, which are likely orphan TcCs encoded at some distance from TcA and TcB homologues. Based on the statistical features of the 2,528 valid TcC proteins identified in previous analysis, 97.2% TcC proteins are of 851–1,050 aa in size and 97.9% TcCs have HVRs of 201–400 aa in size. Therefore, we proposed the following experimental criteria for the screening for potential orphan TcC proteins in complete bacterial genomes that also contain at least one valid Tc locus: (i) the potential orphan TcC protein encodes a RHS core domain but is not in the genomic neighborhood to TcA/TcB; (ii) the size of the putative orphan TcC should be within 851–1,050 aa and (iii) the size of the putative orphan TcC HVR should be within 201–400 aa. With these criteria we successfully identified TcC6 and TcC7 in *P*. *luminescens* as expected, which further confirmed the reliability of these criteria for the identification of orphan TcC homologues.

### Analyses of TcC proteins from EnteroBase

To place the genomes encoding TcC proteins into the context of genetic diversity for the whole *Yersinia* genus, we calculated a phylogeny based on the 1,553 core genes in core genome MLST V1 (cgMLST V1) scheme from all 3,341 genomes deposited in EnteroBase [45]. The sequences of each core gene were aligned using MAFFT. The concatenated, aligned sequences of all core genes were then subjected to neighbor-joining analysis using FastME v2.1 [59]. A similar phylogeny was also calculated on a subset of 5,314 *Salmonella* genomes that was collected to represent the entire genetic diversity of the genus in EnteroBase. The genomes were chosen by either of the three criteria: (1) All 3,228 genomes from non-subspecies I salmonellae, (2) 2,066 representatives of one sequence for each ribosomal MLST ST [66] that contain ≥ 3 genomes in subspecies I, and (3) All 2,995 genomes that carry Tc clusters. A neighbor-joining phylogeny of these 5,314 genomes was then inferred based on the concatenated, aligned sequences of 3,002 core genes in cgMLST scheme V2.

To reveal the finer genetic structure of the TcC proteins in *Salmonella* and *Yersinia*, a preliminary all-against-all comparison was applied on the extracted nucleotides from all the *tcC* genes that encode either the RHS core domain or the HVR region using BLASTn [67]. The results were filtered and only the alignments that covered at least 50% of both sequences were kept and used to draw a histogram (Fig 6A). By visual inspection of the histogram, very few pairs of *tcC* genes shared a nucleotide identity between 76% and 79% in their RHS core domains or HVRs. Therefore each region was subjected to an average-linkage clustering based on its filtered BLASTn result, using the AgglomerativeClustering function in the Python scikit-learn package with linkage = average and distance_threshold = 0.34, which equals to a sequence identity of 76%. The resulted groups (RHS core domain) and sub-clusters (HVR region) were visualized in the neighbor-joining phylogeny for each species in GrapeTree (S6 Fig).

To identify the surrounding genes for each Tc locus, its genomic location were compared with the locations of the core genes that were used in the cgMLST schemes. The core genes that locate most closely to each Tc locus were extracted and compared. The surrounding core genes for Tc loci in the TCR_3, 4 and 5 groups varied within the group. We then visually examined the Tc loci with different surrounding core genes, and found that there are additional transposases around these loci. A designation was then given to each of these transposes based on the family name of its most similar analog in the ISfinder database [68], after a BLASTn comparison using the function implemented in website.

### Additional bioinformatic analysis and Tc database construction

To identify potential conserved protein domains in the C-terminal HVR of TcC, the representative sequences of each HVR clusters were screened using the batch CD-Search service of CDD with an E-value threshold of 0.05 [54]. As comparison, all known bacterial toxins or effectors of type III/IV secretion systems available from the VFDB database were collected and screened for conserved domains with the same method as well [69]. The Sankey analysis was visualized by the SankeyMATIC software.

The T6SS effectors, evolved VgrG proteins are known as a typical PTs family members, some of which are reported to encode known toxin domains [15,70], including CNF1 (PF05785), VIP2 (cl00173), ACD (PF16671) and PG_binding_1 (PF01471). All proteins available from GenBank that are related to these domains were retrieved to the local system individually. Based on the information of CDD, multiple known domains are used in T6SS VgrG proteins, including VgrG (COG3501), T6SS_Vgr (PF13296) and VI_Rhs_Vgr (TIGR03361). Therefore, we produced a non-redundant list of 279,003 proteins that related to at least one of these T6SS VgrG-related domains (accessed at Dec 17, 2019). Then, for each of the aforementioned toxin domains we extracted a subset of proteins that cover both of the toxin and T6SS VgrG-related domains. Finally, the batch CD-Search service of CDD was employed to confirm that a valid evolved VgrG protein should have generally complete (>500 aa) N-terminal T6SS VgrG-related domain as well as a specific C-terminal toxin domain. A total of 23, 41, 450 and 872 qualified evolved VgrG proteins were identified to carry toxin domain of CNF1, VIP2, ACD and PG_binding_1, respectively.

In order to facilitate future studies on the Tc toxin family we constructed a publicly accessible online database, named dbTC (http://www.mgc.ac.cn/dbTC/), to integrate all results generated from previous and current studies. The database employed many of the background MySQL data schema and foreground Perl CGI scripts used in the previous construction of the dbeCIS database [58]. Additional information including HVR clusters, known (or putative) domain functions and the category of potential orphan TcC proteins were stored in a “background” database and presented in dynamic web pages by newly developed CGI scripts for browsing or searching the results.

## Supporting information

S1 Table The details of 171 TcC HVR clusters and the known bacterial toxins or effectors encode the same domain (if exist). (XLSX)

S2 Table. The details of 10 additional TcC HVR clusters from EnteroBase dataset (DOCX)

S1 Fig Phylogenetic Analysis of TcC Protein Candidates. (A) Maximum-likelihood tree of 2,702 identified TcC protein candidates (root on midpoint). Based on protein sequences of RHS core domain, the tree was constructed by FastTree under WAG models with gamma optimization. The minor clade (clade II) excluded from further analysis is highlighted with dotted box. The ten proteins encoding a PAAR domain at the beginning of their N-terminal sequences are indicated by solid dark red circles. Outer bars are color coded by N- and C-termini of TcC proteins in cyan and pink, respectively (to scale). The tree scale represents substitutions per site. (B) Zoom in of the clade I in the phylogenetic tree shown in panel A for a better visualization. The ten proteins encoding a PAAR domain at the beginning of their N-terminal sequences are indicated by solid dark red circles with red branches. (C) Comparison of sequence logos of the C-terminal half (69 sites in multiple alignment) of RHS core domain for the two clades. Clade I and II consist of 174 and 2,528 proteins, respectively. The logos were constructed by WebLogo with default settings and re-numbered to match the residue numbering in TccC2 from *P*. *luminescens*.
(TIF)

S2 Fig Phylogenetic Tree Depicting the Distribution of TcC Proteins in *Proteobacteria*. (A) Bubble chart of bacterial families vs. the percentage of genomes encoding TcC proteins. The size of each bubble is proportional to the number of TcC-encoding genomes identified in each family. (B) Overall distribution of TcC proteins in phylum *Proteobacteria*. Branches with TcC positive bacteria are highlighted in red. The size of blue bubble for each class is proportional to the number of TcC proteins identified in this study. (C) Detailed distribution of TcC proteins in class γ-*Proteobacteria*. Only TcC positive genera are shown for brevity. The green bars on the right indicate the number of TcC proteins identified. Trees are based on the 16S rRNA tree of life from Silva’s Living Tree project (http://www.arb-silva.de/projects/living-tree/).
(TIF)

S3 Fig Cleavage of *P*. *luminescens* TcdB1*-*TccC2. The mutated amino acid residues were colored and underlined in the corresponding sequence (upper panel). The effect of the indicated mutations on auto-proteolysis was analyzed by SDS-PAGE. NTR, N-terminal region; HVR, C-terminal hypervariable region.
(TIF)

S4 Fig The properties of HVRs. Scatterplot showing the distribution range of isoelectric points (*x* axis) and molecular weights (*y* axis) of HVRs of the 2,528 detected TcC proteins. The bacterial families are shown with indicated colors.
(TIF)

S5 Fig Swapping TcC Protein C-terminal HVRs. (A) Negative stain electron micrographs of *P*. *luminescens* TcdA1-TcdB1-TccC2 wild-type holotoxin. Scale bars: 50 nm. (B-C) Translocation of the natural TcC HVRs. The *P*. *luminescens* TcdA1-TcdB1-TccC2 wild-type holotoxin (B) and chimeric holotoxin formed by BC-PTP or BC-CNF1 (C) were incubated for 24 h at pH 8 or pH 11, and then subjected to gel filtration analysis. The fractions corresponding to holotoxin and HVR were analyzed by Western blot with the antibody indicated. (D) Effect of holotoxin formed by TcdA1 and BC-PTP. Cells were seeded into 24-well plates and incubated with 20 nM of holotoxin for 8 h before imaging. Scale bars, 50 μm. Mock, TcdA1 alone. (E) Intoxication of HEK293T cells with holotoxin formed by TcdA1 and BC-CNF1 or the indicated variants. (F) Translocation of the non-natural HVRs. The chimeric holotoxin formed by *P*. *luminescens* TcdA1 and BC-RFP or BC-GFP were examined as described in B. (G-H) Mean fluorescence of holotoxins formed by TcdA1 and BC-RFP or BC-GFP after incubation in pH8 and pH11.
(TIF)

S6 Fig Distribution of RHS Core Domain in *Salmonella* and *Yersinia*. (A-B) The visualization of the seven RHS core groups phylogenies based on concatenated sequences of 5314 selected *Salmonella* genomes (A) or all 3341 *Yersinia* genomes (B) in EnteroBase as shown in Fig 6. The TCRs that are associated with an IS were highlighted in red. (C) One examplar sequence was shown for each TCR group. The genes are color-coded as in the Key. IS elements (orange) are found around TCR_3, 4 and 5.
(TIF)

S7 Fig A Two-level evolutionary model of Tc toxins. (A) A cartoon showing different evolutionary dynamics for 2 of the 3 types of Tc loci described in Fig 7E. Some Tc loci (blue, TCR_1) is not carried by a mobile element, and vertically inherited into the population after gaining at their common ancestor. It also evolutionarily stable and does not acquire new TcC HVRs but only occasionally lost some. In contrast, another Tc loci (orange, TCR_3, 4 & 5) is carried by an IS element, and therefore is able to insert into the populations multiple occasions. It is also evolutionarily active and can acquire new TcC HVRs via homologous recombinations. (B) An assumptive model for TcC replacement. Homologous recombination between two consecutive RHS core encoding regions leads to the generation of a new *tcC* and an episomal circle carrying a HVR region plus the N-terminus of the next *tcC* gene. The episomal circle can then be transferred into a new bacterial cell and incorporated into its Tc locus *via* a second homologous recombination given their sequence identities are sufficiently high, leading to the gain of a new *tcC* gene on that replicon.
(TIF)
