# Supplementary material for: Genome-wide dissection reveals diverse pathogenic roles of bacterial Tc toxins
Source: PLoS Pathog. 2021 Feb 4;17(2):e1009102. doi: 10.1371/journal.ppat.1009102 (PMC7861908; doi:10.1371/journal.ppat.1009102)
Supplement: S2 Table. The details of 10 additional TcC HVR clusters from EnteroBase dataset — (DOCX) [file ppat.1009102.s002.docx]

**Table S2**. The details of 10 additional TcC HVR clusters from EnteroBase dataset.

| **HVR Cluster** | **Putative domain (accession*)** | **Number of TcC proteins** | **Representative bacterial genome (EnteroBase gene ID)** |
| --- | --- | --- | --- |
| TCHC_172 | N/A | 2 | *Salmonella* (SAL_BB4457AA_AS_01574) |
| TCHC_173 | N/A | 5 | *Yersinia* (YER_CA9632AA_AS_02902) |
| TCHC_174 | N/A | 3 | *Yersinia* (YER_CA7987AA_AS_00473) |
| TCHC_175 | N/A | 2 | *Yersinia* (YER_DA2361AA_AS_01852) |
| TCHC_176 | Gly_transf_sug superfamily (cl19952) | 1 | *Yersinia* (YER_CA4372AA_AS_00571) |
| TCHC_177 | N/A | 6 | *Yersinia* (YER_CA8293AA_AS_04303) |
| TCHC_178 | N/A | 2 | *Yersinia* (YER_CA4372AA_AS_00572) |
| TCHC_179 | PRK15386 superfamily (cl26353) | 4 | *Yersinia* (YER_CA9656AA_AS_02690) |
| TCHC_180 | Enterotoxin_a superfamily (cl03779) | 5 | *Yersinia* (YER_CA9632AA_AS_02903) |
| TCHC_181 | N/A | 1 | *Yersinia* (YER_DA2224AA_AS_01059) |

| * Accession No. of NCBI Conserved Domain Database (CDD). N/A, not available. |  |  |  |
| --- | --- | --- | --- |
