# Supplementary figures and images for: Genome-wide dissection reveals diverse pathogenic roles of bacterial Tc toxins
Source: PLoS Pathog. 2021 Feb 4;17(2):e1009102. doi: 10.1371/journal.ppat.1009102 (PMC7861908; doi:10.1371/journal.ppat.1009102)

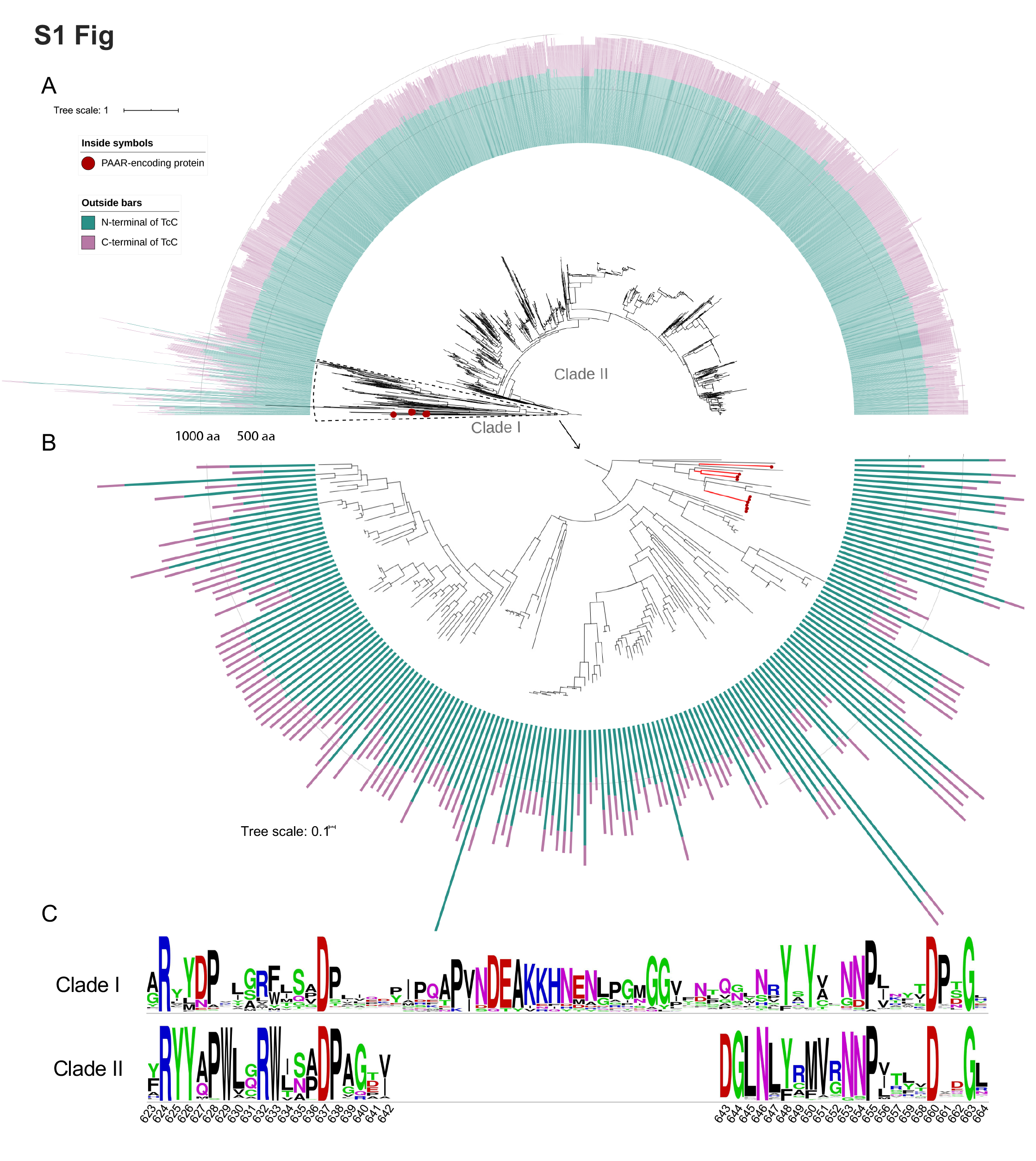

Supplement: S1 Fig — (A) Maximum-likelihood tree of 2,702 identified TcC protein candidates (root on midpoint). Based on protein sequences of RHS core domain, the tree was constructed by FastTree under WAG models with gamma optimization. The minor clade (clade II) excluded from further analysis is highlighted with dotted box. The ten proteins encoding a PAAR domain at the beginning of their N-terminal sequences are indicated by solid dark red circles. Outer bars are color coded by N- and C-termini of TcC proteins in cyan and pink, respectively (to scale). The tree scale represents substitutions per site. (B) Zoom in of the clade I in the phylogenetic tree shown in panel A for a better visualization. The ten proteins encoding a PAAR domain at the beginning of their N-terminal sequences are indicated by solid dark red circles with red branches. (C) Comparison of sequence logos of the C-terminal half (69 sites in multiple alignment) of RHS core domain for the two clades. Clade I and II consist of 174 and 2,528 proteins, respectively. The logos were constructed by WebLogo with default settings and re-numbered to match the residue numbering in TccC2 from P. luminescens. (TIF) [file ppat.1009102.s003.tif]

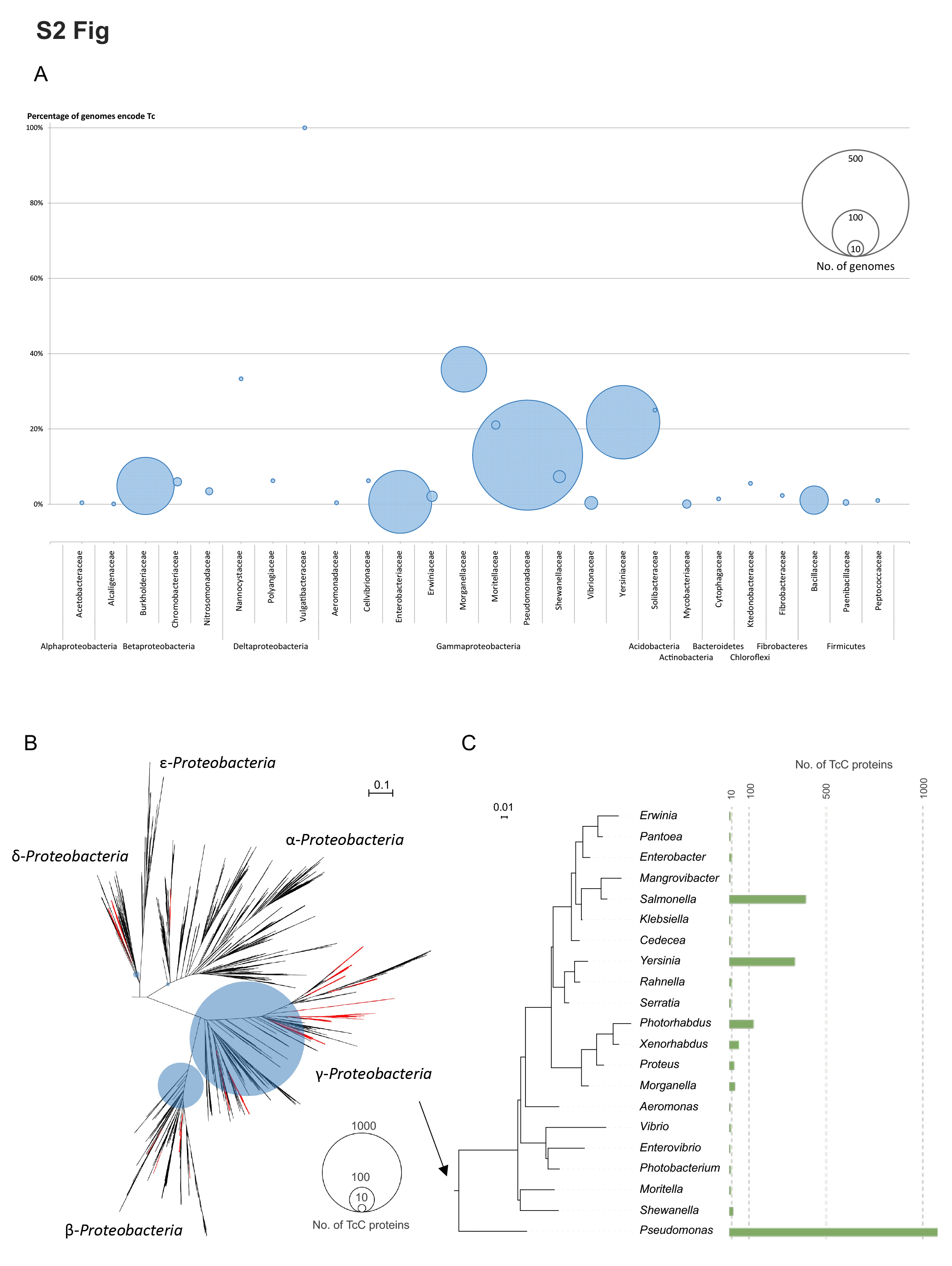

Supplement: S2 Fig — (A) Bubble chart of bacterial families vs. the percentage of genomes encoding TcC proteins. The size of each bubble is proportional to the number of TcC-encoding genomes identified in each family. (B) Overall distribution of TcC proteins in phylum Proteobacteria. Branches with TcC positive bacteria are highlighted in red. The size of blue bubble for each class is proportional to the number of TcC proteins identified in this study. (C) Detailed distribution of TcC proteins in class γ-Proteobacteria. Only TcC positive genera are shown for brevity. The green bars on the right indicate the number of TcC proteins identified. Trees are based on the 16S rRNA tree of life from Silva’s Living Tree project (http://www.arb-silva.de/projects/living-tree/). (TIF) [file ppat.1009102.s004.tif]

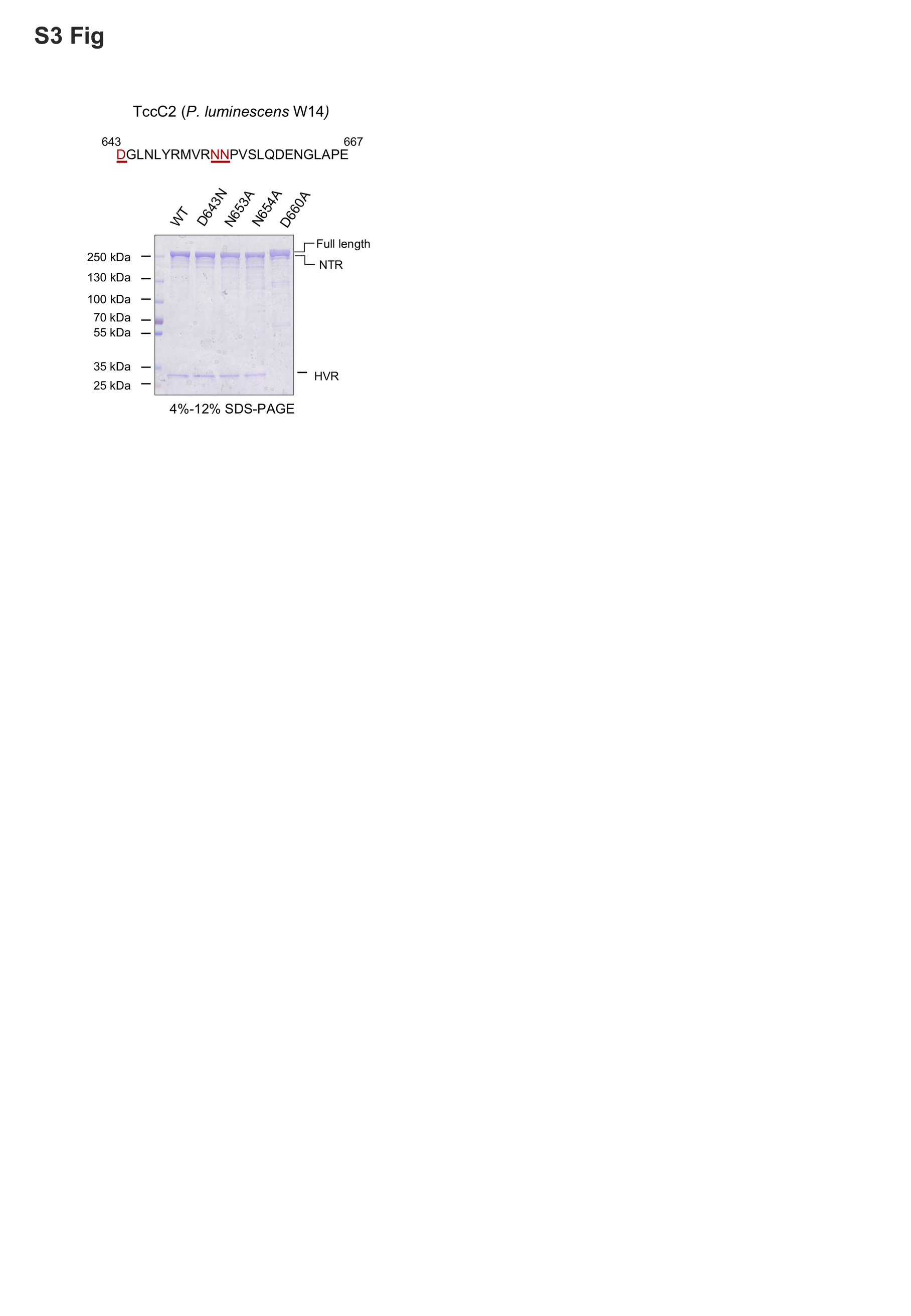

Supplement: S3 Fig — The mutated amino acid residues were colored and underlined in the corresponding sequence (upper panel). The effect of the indicated mutations on auto-proteolysis was analyzed by SDS-PAGE. NTR, N-terminal region; HVR, C-terminal hypervariable region. (TIF) [file ppat.1009102.s005.tif]

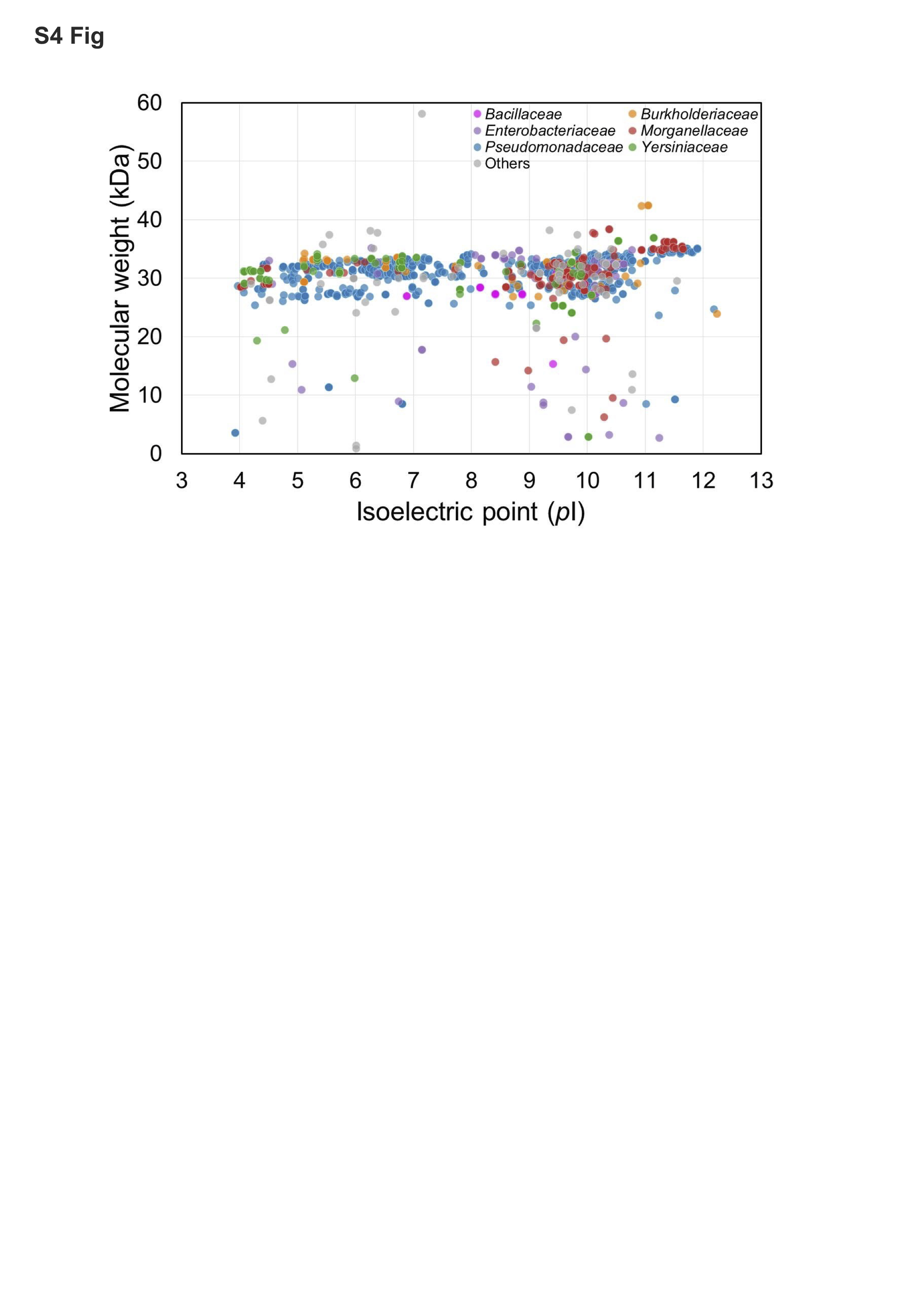

Supplement: S4 Fig — Scatterplot showing the distribution range of isoelectric points (x axis) and molecular weights (y axis) of HVRs of the 2,528 detected TcC proteins. The bacterial families are shown with indicated colors. (TIF) [file ppat.1009102.s006.tif]

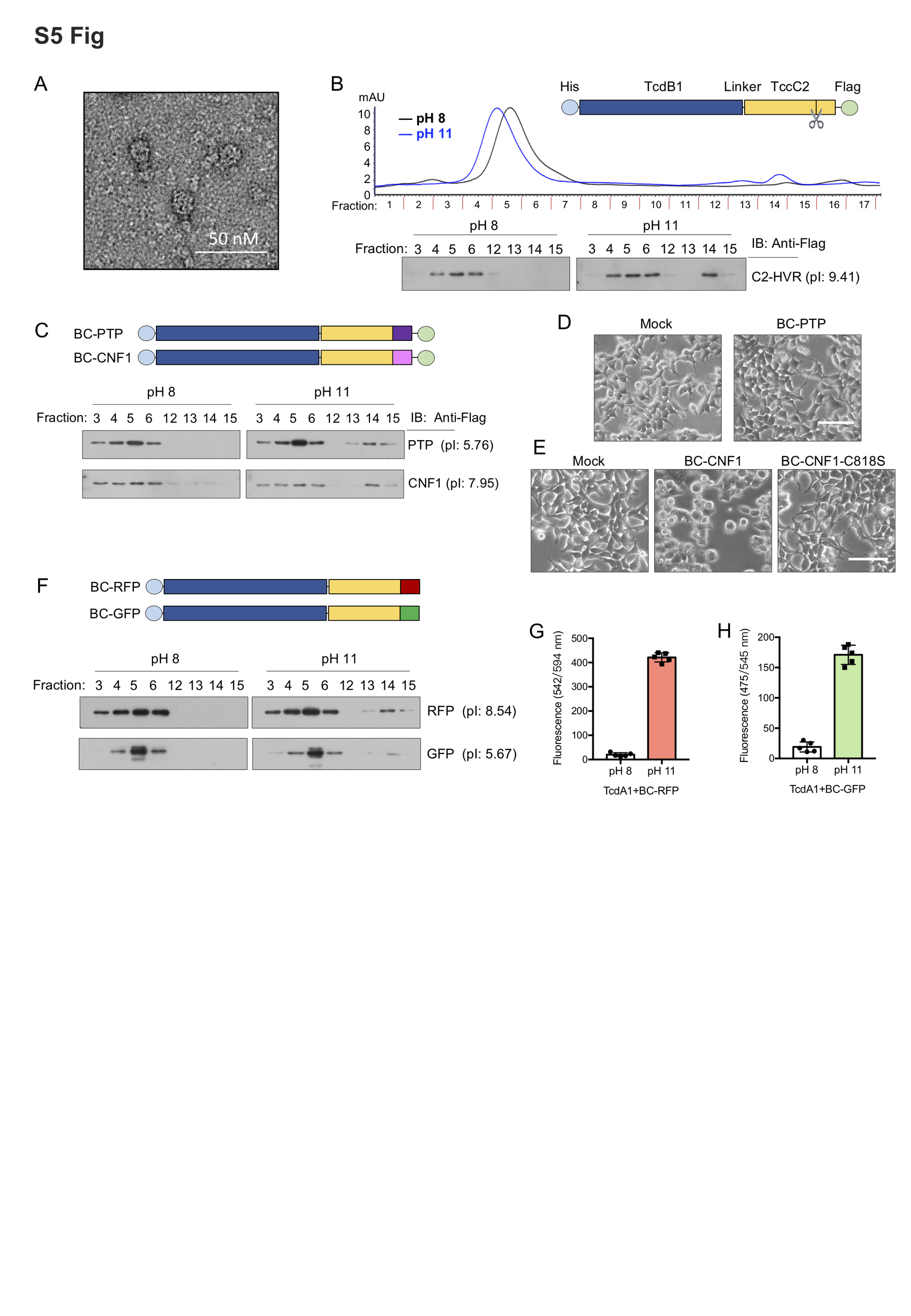

Supplement: S5 Fig — (A) Negative stain electron micrographs of P. luminescens TcdA1-TcdB1-TccC2 wild-type holotoxin. Scale bars: 50 nm. (B-C) Translocation of the natural TcC HVRs. The P. luminescens TcdA1-TcdB1-TccC2 wild-type holotoxin (B) and chimeric holotoxin formed by BC-PTP or BC-CNF1 (C) were incubated for 24 h at pH 8 or pH 11, and then subjected to gel filtration analysis. The fractions corresponding to holotoxin and HVR were analyzed by Western blot with the antibody indicated. (D) Effect of holotoxin formed by TcdA1 and BC-PTP. Cells were seeded into 24-well plates and incubated with 20 nM of holotoxin for 8 h before imaging. Scale bars, 50 μm. Mock, TcdA1 alone. (E) Intoxication of HEK293T cells with holotoxin formed by TcdA1 and BC-CNF1 or the indicated variants. (F) Translocation of the non-natural HVRs. The chimeric holotoxin formed by P. luminescens TcdA1 and BC-RFP or BC-GFP were examined as described in B. (G-H) Mean fluorescence of holotoxins formed by TcdA1 and BC-RFP or BC-GFP after incubation in pH8 and pH11. (TIF) [file ppat.1009102.s007.tif]

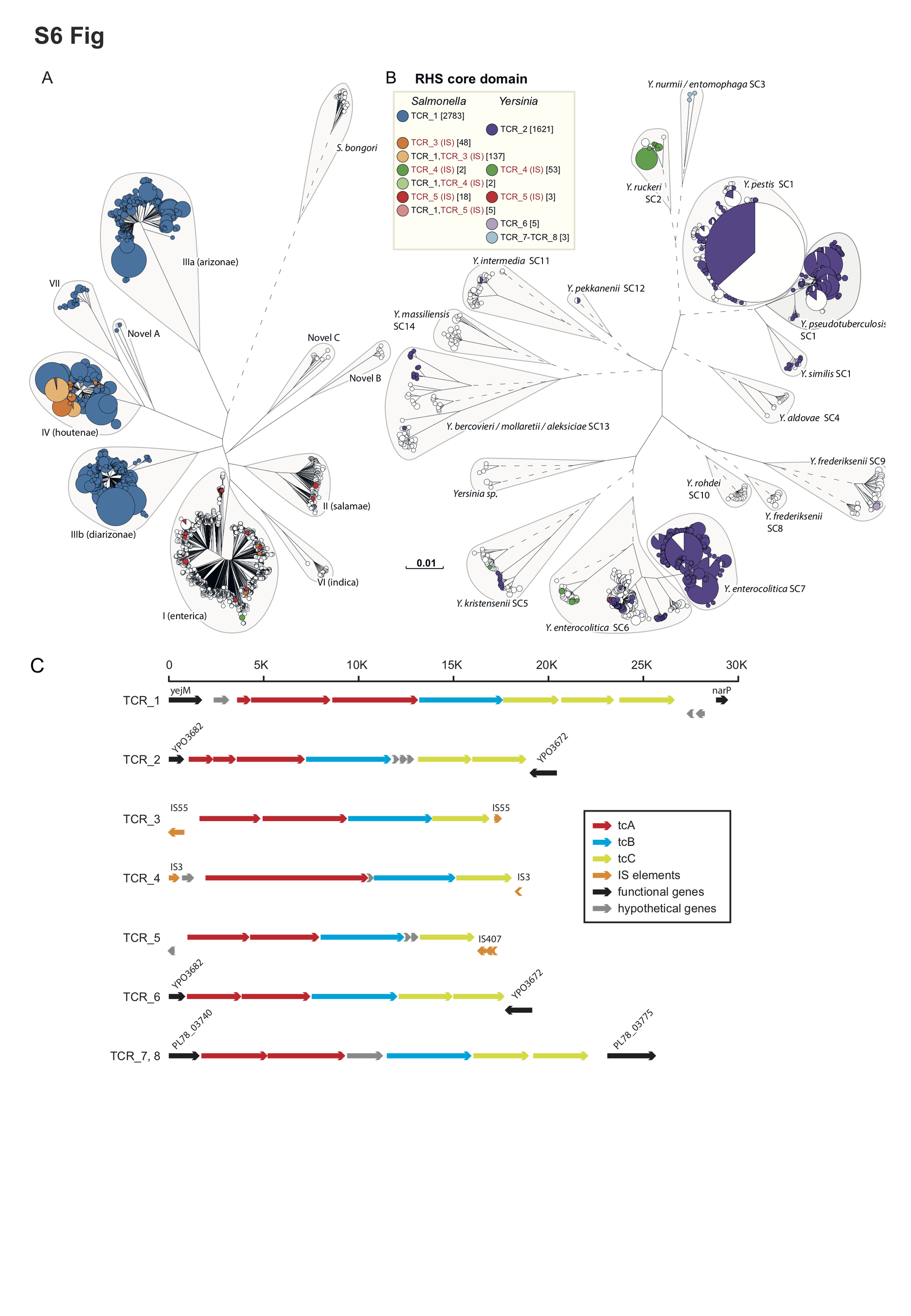

Supplement: S6 Fig — (A-B) The visualization of the seven RHS core groups phylogenies based on concatenated sequences of 5314 selected Salmonella genomes (A) or all 3341 Yersinia genomes (B) in EnteroBase as shown in Fig 6. The TCRs that are associated with an IS were highlighted in red. (C) One examplar sequence was shown for each TCR group. The genes are color-coded as in the Key. IS elements (orange) are found around TCR_3, 4 and 5. (TIF) [file ppat.1009102.s008.tif]

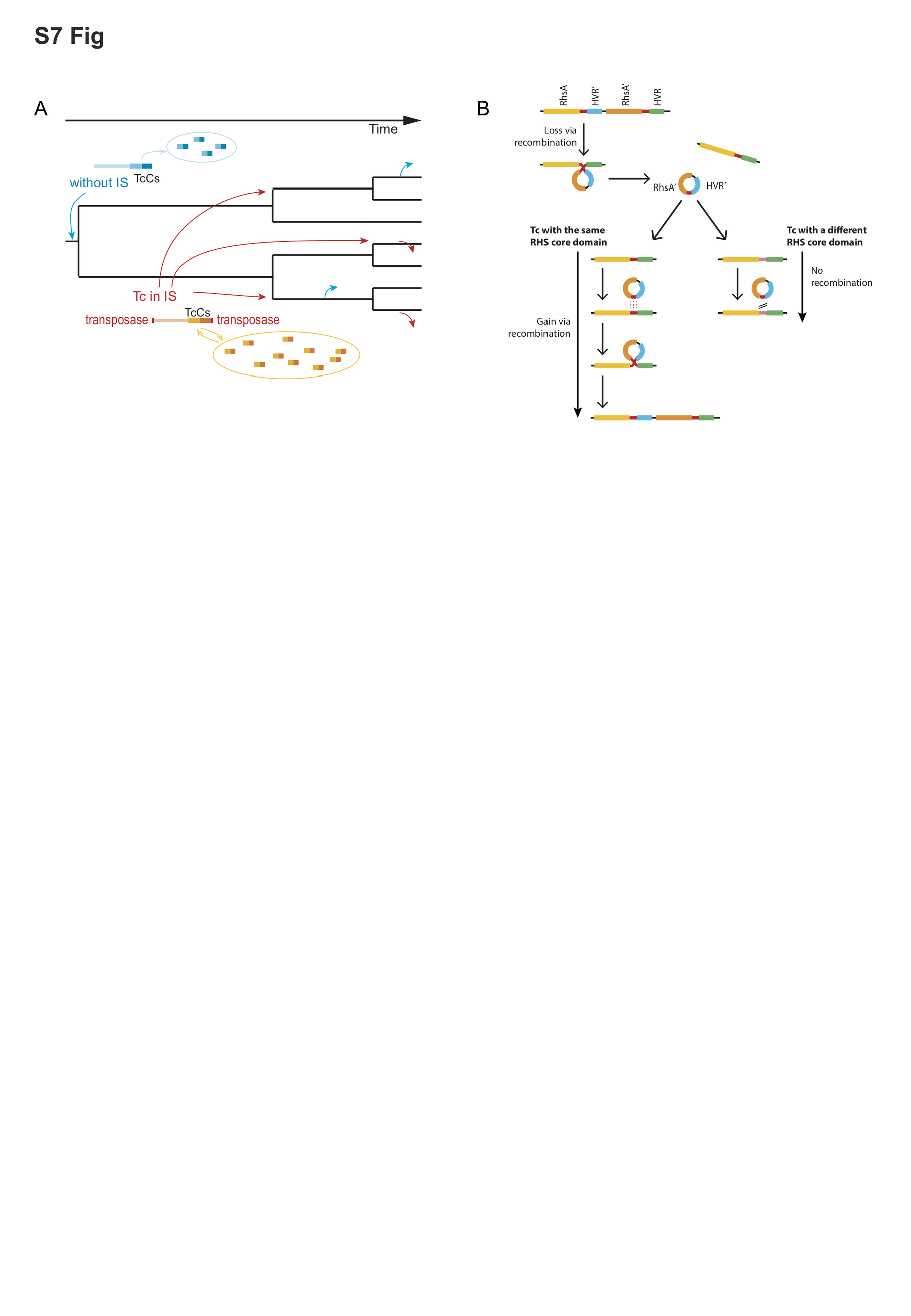

Supplement: S7 Fig — (A) A cartoon showing different evolutionary dynamics for 2 of the 3 types of Tc loci described in Fig 7E. Some Tc loci (blue, TCR_1) is not carried by a mobile element, and vertically inherited into the population after gaining at their common ancestor. It also evolutionarily stable and does not acquire new TcC HVRs but only occasionally lost some. In contrast, another Tc loci (orange, TCR_3, 4 & 5) is carried by an IS element, and therefore is able to insert into the populations multiple occasions. It is also evolutionarily active and can acquire new TcC HVRs via homologous recombinations. (B) An assumptive model for TcC replacement. Homologous recombination between two consecutive RHS core encoding regions leads to the generation of a new tcC and an episomal circle carrying a HVR region plus the N-terminus of the next tcC gene. The episomal circle can then be transferred into a new bacterial cell and incorporated into its Tc locus via a second homologous recombination given their sequence identities are sufficiently high, leading to the gain of a new tcC gene on that replicon. (TIF) [file ppat.1009102.s009.tif]
